# Asymmetry of posterior pole remodeling in high myopia

**DOI:** 10.1038/s41598-026-49683-w

**Published:** 2026-04-21

**Authors:** Ze–xu Wang, Bin Wei, Rui Li

**Affiliations:** 1 Medical College of Wuhan University of Science and Technology, Wuhan, China; 2 Department of Ophthalmology, Xiaogan Hospital, Wuhan University of Science and Technology, the Central Hospital of Xiaogan, Xiaogan, China; 3 The 1st affiliated hospital, Jiangxi Medical College, Nanchang University, Nanchang, Jiangxi China

**Keywords:** Diseases, Health care, Medical research

## Abstract

To characterize posterior pole morphology in high myopia using widefield OCT angiography (OCTA)–derived curvature metrics, with particular emphasis on asymmetry and regional variation, and to determine whether posterior deformation shows structured spatial patterns beyond conventional axial length and refractive measures. Dual–center, cross–sectional observational study. Widefield (24 × 20 mm) OCTA scans were used to derive posterior pole curvature maps. Posterior curvature heterogeneity was quantified using the posterior heterogeneity index (PHI slope), and directional asymmetry was quantified using the nasal–temporal difference in curvature slope (NT diff slope). Linear mixed–effects models accounting for inter–eye correlation were used to compare high myopia (defined as axial length ≥ 26.0 mm or spherical equivalent refraction ≤ − 6.00 D) with non–high myopia, adjusting for age, sex, eye laterality, and study center. Continuous associations with axial length were evaluated, and robustness was assessed across centers and sensitivity analyses. Eyes with discordant axial–refractive phenotypes were analyzed separately. Compared with non–high myopia eyes, high myopia eyes demonstrated significantly greater posterior curvature heterogeneity (PHI slope) and more pronounced nasal–temporal asymmetry (more negative NT diff slope), suggesting a spatially asymmetric pattern of posterior pole curvature. These associations remained significant after adjustment and were consistent across both study centers. Increasing axial length was continuously associated with higher PHI slope and more pronounced nasal–temporal asymmetry. Notably, eyes meeting refractive but not axial criteria for high myopia exhibited posterior pole morphology more similar to concordant high myopia eyes than to non–high myopia eyes, consistent with a structural–refractive dissociation. Key curvature indices showed high short–term repeatability. Posterior pole deformation in high myopia was associated with greater curvature heterogeneity and more pronounced nasal–temporal asymmetry, rather than a uniformly distributed pattern. Widefield OCTA–derived curvature metrics may provide a reproducible and clinically interpretable framework that complements conventional axial length and refractive assessment, particularly in eyes with discordant structural and refractive characteristics. Widefield OCTA–derived curvature mapping provides quantitative, reproducible descriptors of posterior pole remodeling that complement conventional measures of myopia severity (axial length and refraction). These interpretable morphology indices may help describe and compare structural variation across eyes with different axial–refractive profiles, particularly in those with discordant axial–refractive characteristics.

**Supplementary Information:**

The online version contains supplementary material available at 10.1038/s41598-026-49683-w.

## Introduction

High myopia is a major cause of irreversible visual impairment worldwide and is associated with posterior segment complications such as myopic maculopathy, posterior staphyloma, and tractional maculopathies^1–4^. Clinically, high myopia is commonly defined and stratified by axial length (AL) and spherical equivalent refraction (SER)^3,5^. Yet eyes with comparable AL or refractive error can exhibit markedly different posterior pole configurations and clinical trajectories, suggesting that conventional global metrics do not fully capture structural variation in myopia^6–8^.

Posterior pole deformation is a hallmark of high myopia. Prior work using fundus imaging, optical coherence tomography (OCT), and magnetic resonance imaging (MRI) has shown that myopic eyes often demonstrate steepened and irregular posterior contours with regional predilection^6,9–12^. However, most existing approaches are limited by qualitative classification, localized measurements, or reliance on regional mean curvature, which restricts the ability to characterize posterior pole shape across a broad area and to quantify directional organization^6,9,10^.

Widefield OCT and OCT angiography (OCTA) now enable large–area imaging of the posterior pole, creating an opportunity to quantify curvature across central and more peripheral regions^13^. Curvature–based analyses have been proposed to describe posterior pole shape and irregularity, but key gaps remain. First, it is unclear whether posterior pole deformation in high myopia is best described as nonspecific irregularity or as a reproducible pattern of spatial asymmetry^9,10^. Second, the relationship between posterior pole morphology and conventional measures of myopia severity—particularly in eyes with discordant AL and SER—remains incompletely defined^6,7,10^. Third, the robustness and repeatability of curvature–derived morphology indices across centers and analytic strategies have not been systematically evaluated^14–16^.

These gaps are clinically relevant. In routine practice, clinicians frequently encounter eyes with substantial refractive myopia but sub–threshold axial elongation, and conversely, elongated eyes with limited refractive error^6,7^. In such discordant cases, AL and SER alone provide limited structural context for posterior pole configuration. A quantitative and interpretable description of posterior pole morphology could therefore complement conventional metrics without redefining diagnostic thresholds.

In this dual–center study, we quantified posterior pole morphology using curvature metrics derived from widefield OCTA imaging. We focused on two interpretable indices: posterior curvature heterogeneity (PHI slope) and nasal–temporal asymmetry (NT diff slope), reflecting non–uniformity and directional bias of posterior remodeling, respectively. Using linear mixed–effects models accounting for inter–eye correlation, we compared high myopia and non–high myopia eyes, evaluated continuous associations with axial length, and examined morphology in discordant AL–SER phenotypes. We additionally assessed robustness across centers and sensitivity analyses and evaluated short–term repeatability of key indices.

By extending assessment beyond global axial elongation to posterior pole asymmetry, this study aims to provide a quantitative framework for describing posterior pole morphology in high myopia and to clarify how curvature–derived metrics may complement conventional clinical measures.

## Methods

### Study population

This dual–center observational study was conducted at Xiaogan Central Hospital and the First Affiliated Hospital of Nanchang University. The study adhered to the principles of the Declaration of Helsinki, and the study protocol was approved by the Institutional Review Board/Ethics Committee (approval number: KY–2025102201). Written informed consent was obtained from all participants prior to enrollment.

Inclusion criteria comprised age 20–40 years, bilateral intraocular pressure (IOP) ≤ 21 mmHg, refractive error of − 6.00 D < SER ≤ − 0.50 D for non–high myopia participants, or SER ≤ − 6.00 D or axial length ≥ 26 mm for high myopia participants, best–corrected visual acuity (BCVA) ≥ 0.8, image quality score ≥ 8, and voluntary informed consent to participate. Exclusion criteria included any ocular disease besides myopia (e.g., cataracts, glaucoma, diabetic retinopathy, uveitis), prior retinal laser treatment, or ocular surgery history. Participants with systemic chronic diseases (e.g., diabetes, hypertension, hyperthyroidism) were excluded. Exclusion criteria also included ocular media opacities impairing high–quality imaging, motion artifacts, and layered artifacts uncorrectable by manual adjustment.

### Ophthalmic examination and imaging

All participants underwent a series of ophthalmic examinations, including medical history collection, intraocular pressure measurement, cycloplegic refraction, slit–lamp biomicroscopy, dilated fundus examination, measurement of ocular biometric parameters, and SS–OCT/OCT imaging. Pupil dilation was achieved using 1% tropicamide eye drops administered three times at 5–minute intervals before refraction and imaging. Biometric parameters such as AL, CCT, ACD, VT, and Km were obtained using an ophthalmic biometer. SS–OCT and OCT angiography (OCTA) scans were acquired using a 400 kHz swept–source OCTA system (BM–400 K BMizar, Tupai Medical Technology, Beijing, China) with a 1060–nm vertical–cavity surface–emitting laser (VCSEL), scanning speed of 400,000 A–scans/s, axial resolution of 3.8 μm, and lateral resolution of 10 μm. A single ultra–widefield 24 × 20 mm scan centered on the fovea (1536 A–scans × 1280 B–scan positions; A–scan depth 6.0 mm [2560 pixels]) was obtained for each eye, providing a field of view of up to 120 degrees; two consecutive B–scans were acquired at each location to support flow detection and improve signal stability. Before curvature quantification, OCT volumes underwent motion artifact reduction and manual review, and Bruch’s membrane segmentation was verified and corrected when necessary. A fitted three-dimensional retinal surface model was generated based on the reconstructed Bruch’s membrane, and curvature was quantified as Gaussian curvature (K) at each point across the full 24 × 20 mm region (1536 × 1280 points). Axial length–based magnification correction was applied during image processing. No additional correction based on corneal curvature was applied.

### Posterior pole curvature mapping

**Fig. 1:**
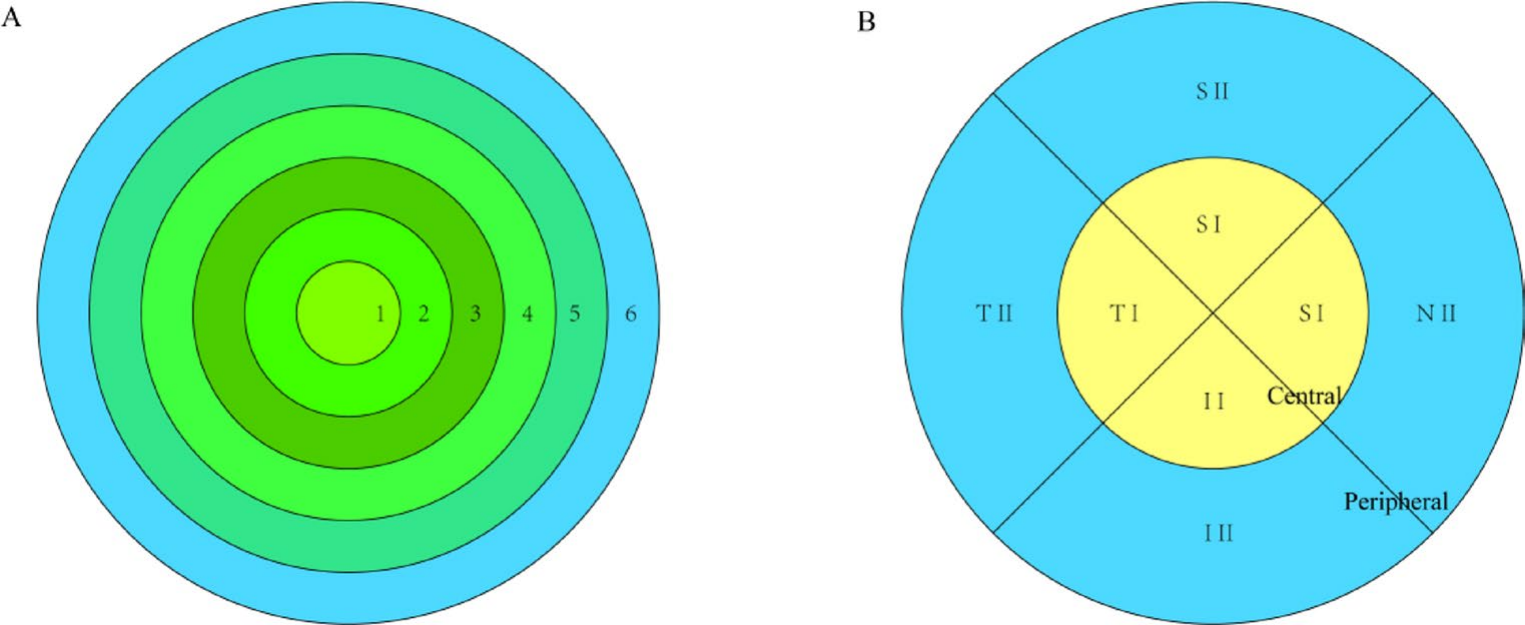
Schematic illustration of posterior pole curvature analysis. (**A**) Ring-based segmentation of the posterior pole (R1–R6). (**B**) Quadrant-based subdivision into superior (S), inferior (I), nasal (N), and temporal (T) sectors. Schematic of the spatial framework for curvature-derived metrics.

Posterior pole curvature was calculated from three–dimensional OCT structural data using a curvature–based surface modeling approach. The posterior retinal surface was segmented and fitted to generate curvature maps across the scanned area. The posterior pole was divided into six concentric rings (R1–R6) centered on the fovea, with outer diameters of 1, 3, 6, 9, 12, and 15 mm, respectively, extending from the central macula to the peripheral posterior pole. Because the innermost ring (R1) corresponds to the foveal region and cannot be reliably subdivided into quadrants, quadrant–specific analyses were performed using rings R2–R6. Curvature values were calculated for each ring and for four anatomical quadrants (superior, inferior, nasal, and temporal) where applicable (Fig. 1).

### Curvature–derived morphology metrics

Several curvature–derived metrics were calculated to characterize posterior pole morphology:

Posterior Curvature Heterogeneity (PHI slope): PHI slope was defined as the standard deviation of quadrant–specific posterior pole curvature slopes in the superior (S), inferior (I), nasal (N), and temporal (T) directions (PPslope–S, PPslope–I, PPslope–N, and PPslope–T), reflecting the degree of directional heterogeneity of posterior pole remodeling. Directional Asymmetry (NT diff slope): NT diff slope was defined as the difference between temporal and nasal posterior pole curvature slopes (PPslope T − PPslope N), reflecting systematic nasal–temporal asymmetry. Posterior Pole Curvature Slope (PPslope): For each quadrant, PPslope was calculated as the slope of the linear regression between curvature values and concentric ring index (R2–R6), analogous to peripheral corneal steepening, representing curvature change per unit radial distance. Posterior Expansion Indices: Posterior expansion indices (PEI and dPEI) were calculated as differences between outer and inner ring curvature values, with negative values indicating increased posterior expansion toward the periphery. Quadrant–specific dPEI values were calculated where applicable. A detailed description of all curvature metrics and formulas is provided in the Supplementary Methods.

### Repeatability and reliability

All SS–OCT scans were performed by an experienced technician following standardized procedures. Each subject underwent examination in a dark room between 2:00 PM and 5:00 PM after resting quietly for 15 min prior to the examination. Short–term repeatability of PHI slope and NT diff slope was assessed in a subset of eyes that underwent repeated OCTA imaging across two consecutive days. Each eye was scanned three times on the first day and once on the second day. Intraclass correlation coefficients (ICC, two–way random–effects model, absolute agreement) were calculated to evaluate repeatability. Detailed results are provided in the Supplementary Material.

### Statistical analysis

Analyses were conducted at the eye level. Therefore, each eye was classified independently as HM or non–HM according to its own axial length and spherical equivalent refraction. HM was modeled as a binary variable (HM vs. non–HM), and participants could contribute one HM eye and one non–HM eye. For participant-level descriptive analyses, one row per participant was constructed. For eye-level continuous variables, participant-level summaries were derived by averaging values across both eyes, and high myopia status was assigned using participant-level axial length and spherical equivalent refraction summaries. For the prespecified primary endpoint (PHI slope) and key secondary endpoint (NT diff slope), we fitted linear mixed–effects models (LMMs) of the form: morphology metric ~ HM + age + sex + eye laterality + study center + (1 | subject ID), where the morphology metric was the dependent variable. Continuous associations with myopia severity were evaluated using analogous models replacing HM with axial length (AL) as the primary predictor; AL and spherical equivalent refraction (SER) were not included simultaneously in the same model. Generalizability across centers was assessed by fitting identical models within each center and by testing effect modification using an HM × center interaction term. Sensitivity analyses included generalized estimating equations (GEE) with an exchangeable correlation structure and clustering by subject ID, analyses restricted to one eye per participant, alternative definitions of high myopia, and trimming or winsorization to assess robustness to extreme values. For secondary or exploratory curvature–related outcomes, multiplicity was controlled using the Benjamini–Hochberg false discovery rate procedure. All analyses were performed in R (version 4.5.1; R Foundation for Statistical Computing, Vienna, Austria). Two–sided P values < 0.05 were considered statistically significant unless otherwise specified.

## Results

### Study population and baseline characteristics

A total of 143 participants (286 eyes) were included in the final analysis. For participant-level descriptive analyses, one row per participant was constructed. Baseline characteristics of the study population are summarized in Table 1.

**Table 1:** Baseline characteristics by high myopia status.

Characteristic	Total (N = 143)	Non-HM (N = 73)	HM (N = 70)	value
Age	34.00[25.00, 40.00]	33.00[24.00, 38.00]	34.50[27.00, 41.00]	0.265
Sex				0.716
Female	91.0 (63.6%)	48.0 (65.8%)	43.0 (61.4%)	
Male	52.0 (36.4%)	25.0 (34.2%)	27.0 (38.6%)	
Center				0.014
Center 1	91.0 (63.6%)	54.0 (74.0%)	37.0 (52.9%)	
Center 2	52.0 (36.4%)	19.0 (26.0%)	33.0 (47.1%)	
Axial Length (mm)	25.36[24.48, 26.35]	24.50[23.96, 25.13]	26.37[25.92, 27.17]	<0.001
SER (D)	-5.38[-7.13, -3.00]	-3.13[-4.38, -1.75]	-7.25[-9.50, -6.38]	<0.001
IOP (mmHg)	15.07 ± 2.28	14.80 ± 2.24	15.35 ± 2.30	0.150
CCT (µm)	533.28 ± 31.39	532.13 ± 29.97	534.48 ± 32.98	0.657
ACD (mm)	3.70[3.48, 3.89]	3.66[3.43, 3.86]	3.73[3.58, 3.90]	0.069
Vitreous Thickness (mm)	17.80[16.99, 18.72]	16.99[16.54, 17.60]	18.72[18.19, 19.45]	<0.001
AL/CR Ratio	3.27 ± 0.18	3.15 ± 0.12	3.39 ± 0.14	<0.001
K1 (D)	42.95 ± 1.30	43.04 ± 1.26	42.87 ± 1.36	0.437
K2 (D)	43.69 ± 1.33	43.70 ± 1.18	43.68 ± 1.48	0.934
Km (D)	43.32 ± 1.30	43.36 ± 1.20	43.27 ± 1.40	0.680
WTW (mm)	11.76 ± 0.38	11.75 ± 0.34	11.78 ± 0.41	0.563

Baseline demographic and ocular characteristics for the overall cohort and stratified by high myopia (HM) status. HM was defined as axial length (AL) ≥ 26.0 mm or spherical equivalent refraction (SER) ≤ −6.00 D. For participant-level descriptive analyses, one row per participant was constructed. For eye-level continuous variables, participant-level summaries were derived by averaging values across both eyes. Continuous variables are presented as mean ± SD or median [interquartile range], and categorical variables as n (%). P values are from unadjusted comparisons between HM and non-HM groups at the participant level (Welch t-test, Wilcoxon rank-sum test, or Pearson χ² test, as appropriate). Primary inferential analyses were conducted at the eye level and accounted for within-subject correlation using mixed-effects models.

ACD = anterior chamber depth; AL = axial length; AL/CR = axial length–to–corneal radius ratio; CCT = central corneal thickness; D = diopters; HM = high myopia; IOP = intraocular pressure; K1 = flat keratometry; K2 = steep keratometry; Km = mean keratometry; SER = spherical equivalent refraction; WTW = white-to-white corneal diameter.

### Posterior pole morphology differs between high myopia and non–high myopia

**Fig. 2:**
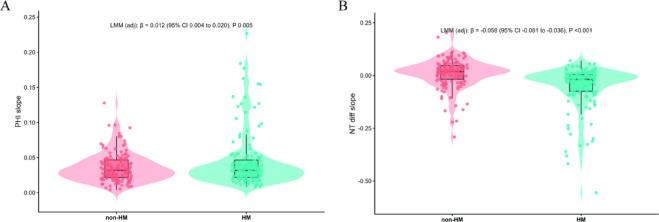
Posterior pole morphology differs between high myopia and non–high myopia. (**A**) Distribution of PHI slope by high myopia (HM) status. (**B**) Distribution of NT diff slope by HM status. Violin plots show eye-level distributions with overlaid data points and boxplots (median and interquartile range). Estimates are from linear mixed-effects models (LMMs) adjusted for age, sex, eye laterality, and study center, with subject-specific random intercepts. β, 95% confidence intervals (CI), and P values are shown in each panel. Abbreviations: CI = confidence interval; HM = high myopia; LMM = linear mixed-effects model; NT = nasal–temporal; PHI = posterior heterogeneity index.

Primary endpoint: posterior curvature heterogeneity (PHI slope). Posterior curvature heterogeneity, quantified by PHI slope, was significantly greater in eyes with high myopia than in non–HM eyes (Fig. 2A). In linear mixed–effects models adjusted for age, sex, eye laterality, and center with subject–level random effects, high myopia was associated with a higher PHI slope (β = 0.012, 95% CI 0.004 to 0.020, *P* = 0.005) (Table 2).

**Table 2:** Association of high myopia status with posterior pole morphology metrics.

index	β	95% CI	*P* value	*P* FDR
Primary index				
PHI slope	0.012	0.004 to 0.020	**0.005**	—
Key secondary index				
NT diff slope	−0.058	−0.081 to − 0.036	**< 0.001**	—
Other key indicators				
RC6	−0.357	−0.473 to − 0.241	**< 0.001**	**< 0.001**
RC Peripheral	−0.526	−0.816 to − 0.235	**< 0.001**	**< 0.001**
PPslope T	−0.051	−0.072 to − 0.030	**< 0.001**	**< 0.001**
dPEI T	0.189	0.112 to 0.266	**< 0.001**	**< 0.001**
PHI dPEI	0.04	0.012 to 0.068	**0.005**	**0.005**

β represents the adjusted mean difference between HM and non–HM eyes derived from linear mixed-effects models of the form morphology metric ~ HM + age + sex + eye laterality + study center + (1 | subject ID). P values are two-sided. *Benjamini–Hochberg false discovery rate (BH-FDR) correction was applied only to the “Other key metrics” (not to the primary or key secondary outcomes).

HM = high myopia; PHI = posterior heterogeneity index; NT diff = nasal–temporal difference; RC = retinal curvature; dPEI = directional posterior expansion index.

Key secondary endpoint: nasal–temporal asymmetry (NT diff slope). Directional asymmetry of posterior pole curvature, quantified by NT diff slope, also differed significantly between groups. High myopia was associated with a more negative NT diff slope compared with non–HM eyes (β=−0.058, 95% CI − 0.081 to − 0.036, *P* < 0.001), suggesting more pronounced nasal–temporal asymmetry in posterior pole morphology (Fig. 2B; Table 2).

Additional posterior pole morphology metrics. Several additional curvature–based metrics demonstrated significant differences between HM and non–HM eyes after adjustment (Table 2). High myopia was associated with lower curvature values in peripheral and outer ring regions (RC6 and RC peripheral), as well as lower posterior pole slope in the temporal quadrant (PPslope T). In contrast, posterior expansion indices (dPEI T) and directional heterogeneity measures (PHI dPEI) were significantly higher in HM eyes. False discovery rate (FDR) correction for secondary metrics confirmed the robustness of these associations (all adjusted *P* < 0.05).

### Quadrant–specific remodeling patterns

**Fig. 3:**
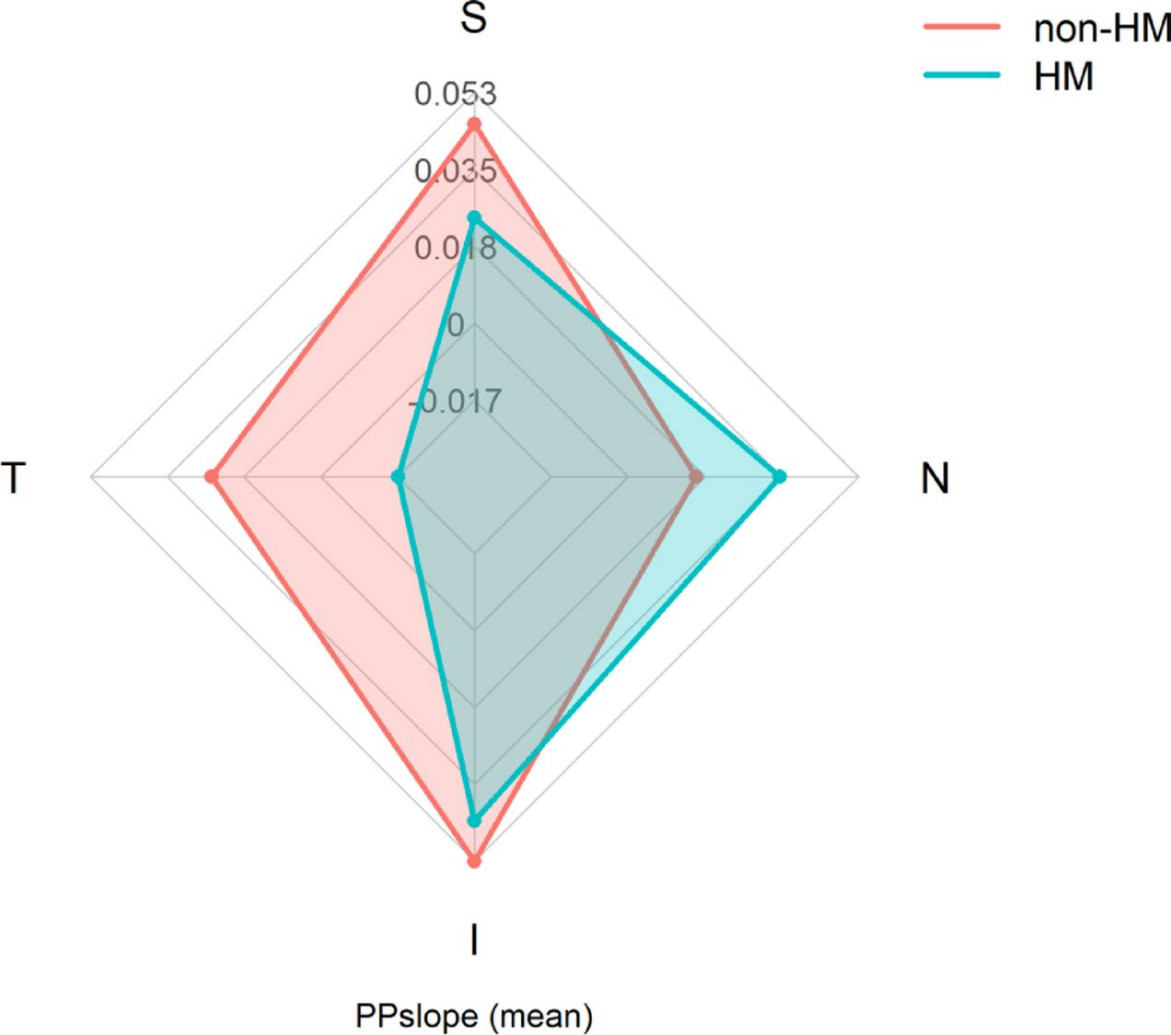
Quadrant pattern of posterior pole curvature slope (PPslope) by high myopia status. Radar plots show group mean posterior pole curvature slope (PPslope) in the superior (S), inferior (I), nasal (N), and temporal (T) quadrants for high myopia (HM) and non–high myopia (non–HM) eyes. Abbreviations: HM = high myopia; non–HM = non–high myopia; PPslope = posterior pole curvature slope.

Quadrant–level analysis revealed distinct spatial patterns of posterior pole remodeling. Radar plots of posterior pole slope (PPslope) demonstrated asymmetric deformation across superior, inferior, nasal, and temporal quadrants, with group–specific differences in the relative contribution of each quadrant (Fig. 3).

A similar but complementary pattern was observed for quadrant–specific expansion indices (dPEI), presented in the Supplementary Materials (Supplementary Figure S12). Together, these analyses suggest that posterior pole deformation in high myopia may show a spatially asymmetric pattern rather than a uniformly distributed one.

### Continuous associations with axial length

**Fig. 4:**
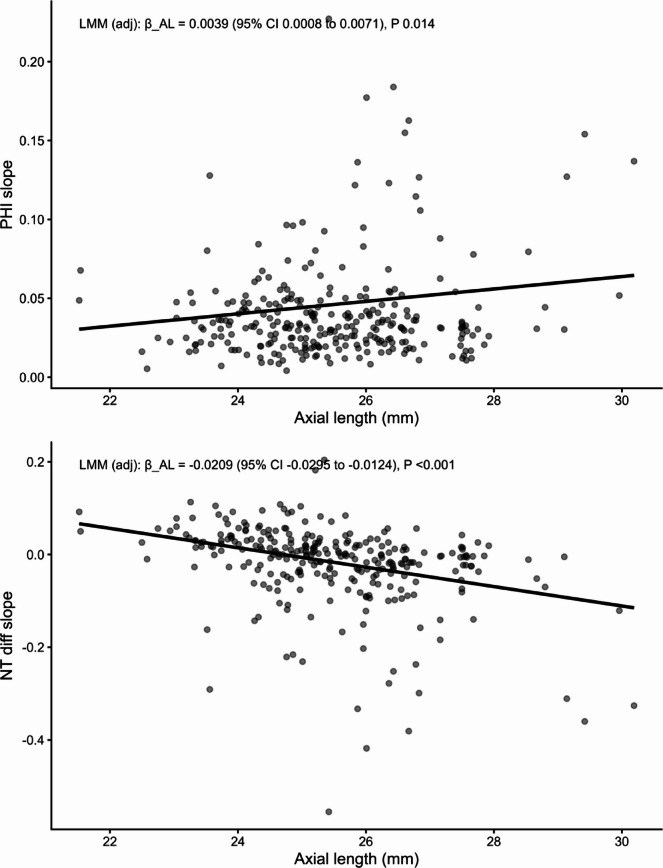
Continuous association between axial length and posterior pole morphology metrics. Scatterplots show eye-level associations between axial length (AL) and posterior pole morphology metrics: (top) PHI slope and (bottom) NT diff slope. Solid lines represent fitted linear trends from linear mixed-effects models (LMMs) adjusted for age, sex, eye laterality, and study center, with subject-specific random intercepts. β, 95% confidence intervals (CI), and P values are shown in each panel. Abbreviations: AL = axial length; CI = confidence interval; LMM = linear mixed-effects model; NT = nasal–temporal; PHI = posterior heterogeneity index.

Axial length showed continuous associations with posterior pole morphology across the full spectrum of myopia severity. Increasing AL was associated with higher PHI slope (β AL = 0.0039, 95% CI 0.0008 to 0.0071, *P* = 0.014) and more negative NT diff slope (β AL = − 0.0209, 95% CI − 0.0295 to − 0.0124, *P* < 0.001) in adjusted mixed–effects models (Fig. 4).

### Center–specific effects and generalizability

**Fig. 5:**
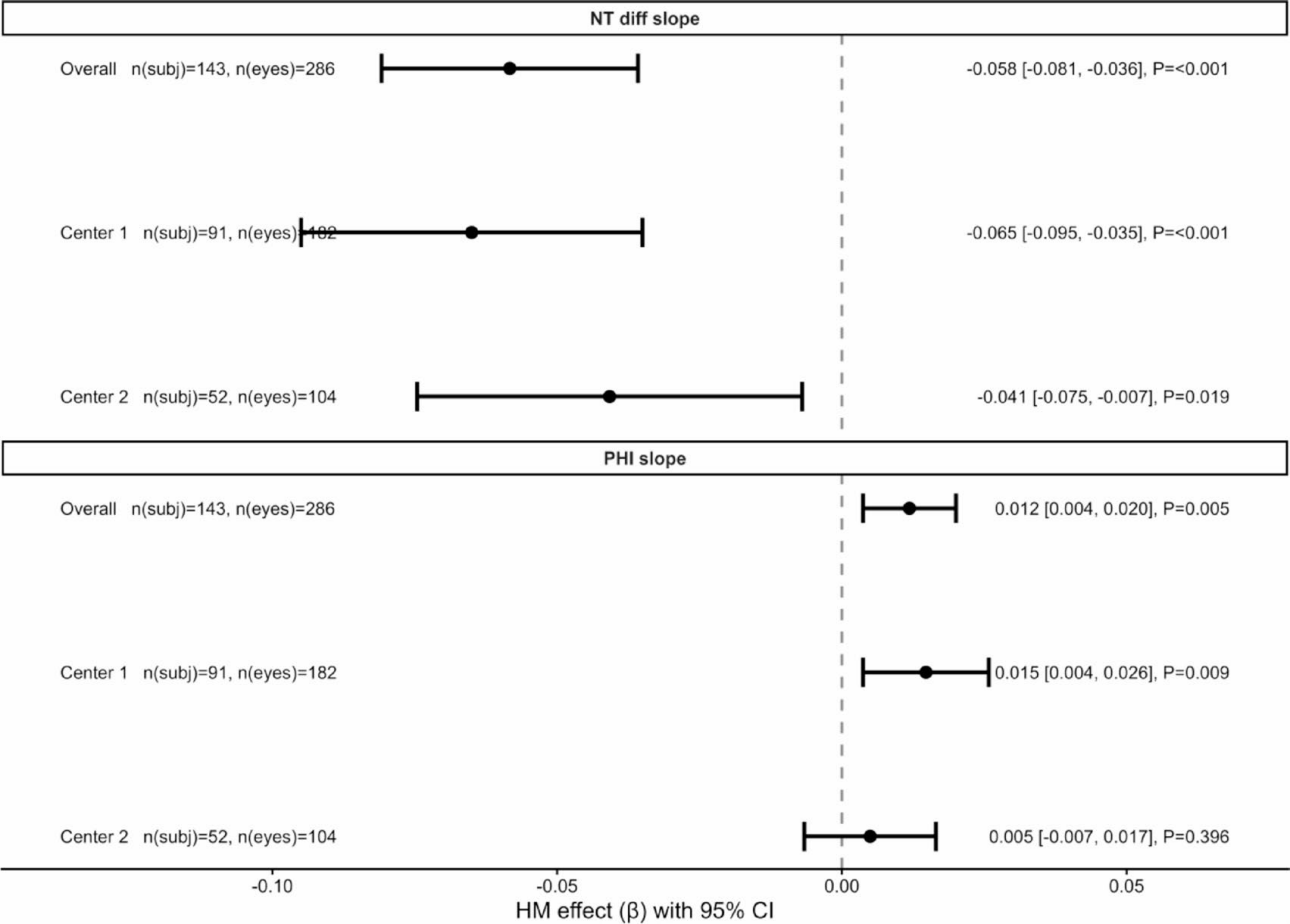
Center-specific effects of high myopia on posterior pole morphology. Forest plots show center-stratified and overall associations between high myopia (HM) and posterior pole morphology metrics: NT diff slope (top) and PHI slope (bottom). Points represent adjusted regression coefficients (β) from linear mixed-effects models (LMMs), with horizontal bars indicating 95% confidence intervals (CIs). The vertical dashed line denotes the null (β = 0). Models were adjusted for age, sex, eye laterality, and study center, with subject-specific random intercepts. Abbreviations: β = regression coefficient; CIs = confidence intervals; HM = high myopia; LMM = linear mixed-effects model; NT = nasal–temporal; PHI = posterior heterogeneity index.

Center–stratified analyses demonstrated consistent directions of structural effects across centers. Forest plots of center–specific estimates showed overlapping confidence intervals for both PHI slope and NT diff slope, with no evidence of qualitative heterogeneity between centers (Fig. 5).

### Sensitivity analyses and model robustness

Results were robust across multiple sensitivity analyses. Analyses restricted to one eye per participant (right eye or randomly selected eye), alternative modeling using generalized estimating equations, and center–standardized z–score comparisons yielded consistent effect directions and magnitudes (Supplementary Figures S2–S4). Model diagnostics demonstrated acceptable residual distributions and no major violations of model assumptions (Supplementary Figure S5). Trimming and winsorization to address potential outliers did not materially alter the results (Supplementary Figure S6). Alternative definitions of high myopia (AL–only versus SER–only) yielded consistent patterns of posterior pole morphology differences (Supplementary Figure S7).

### Structural–refractive discordance phenotypes

Eyes with discordant AL–SER phenotypes exhibited posterior pole morphology patterns distinct from concordant non–HM eyes. In planned contrast analyses, SER–only high myopia eyes demonstrated PHI slope and NT diff slope values closer to concordant HM eyes than to concordant non–HM eyes (Supplementary Figure S20), despite not meeting axial length criteria for high myopia. These findings indicate that posterior pole morphology captures structural features not fully explained by refractive or axial classifications alone.

### Repeatability of posterior pole morphology metrics

Short–term repeatability analyses based on repeated measurements across two centers demonstrated high reproducibility for both PHI slope and NT diff slope. Intraclass correlation coefficients exceeded 0.90 for all metrics and were above 0.97 for both indices, indicating excellent repeatability (Supplementary Table S1).

## Discussion

In this study, we performed a comprehensive, curvature–based analysis of posterior pole morphology in eyes with and without high myopia using widefield OCT–derived metrics. We found that posterior pole deformation in high myopia was associated not only with greater global curvature heterogeneity but also with more pronounced asymmetry, particularly along the nasal–temporal axis. Importantly, these structural features were consistently observed across centers, robust to multiple sensitivity analyses, and reproducible on repeated measurements^23^.

A central finding of this study is that posterior pole deformation in high myopia may not be uniformly distributed across directions. While high myopia was associated with greater overall heterogeneity of posterior curvature (as reflected by higher PHI slope), it was also characterized by a systematic nasal–temporal asymmetry, quantified by a more negative NT diff slope. These findings suggest that posterior pole remodeling in high myopia may not be uniformly distributed across directions and may instead show preferential spatial asymmetry^17,21,26^. This observation is consistent with clinical experience that myopic fundus changes often exhibit regional predilection, but extends prior qualitative observations by providing a quantitative and reproducible framework^19,20,22^.

Previous studies have used magnetic resonance imaging or cross–sectional OCT images to characterize posterior staphyloma and fundus curvature, demonstrating that eyes with myopic maculopathy often exhibit steeper and more irregular posterior contours. However, these approaches have been limited by restricted spatial coverage, high cost, or reliance on qualitative classification^18,23^. Our study differs in several important aspects. First, by leveraging widefield OCT–based curvature mapping, we were able to quantify posterior pole morphology across a large retinal area, capturing both central and peripheral regions. Second, rather than relying on single–point curvature or average curvature alone, we decomposed posterior pole morphology into interpretable components reflecting heterogeneity, directional bias, and regional expansion^22,23^. This approach allows for a more nuanced description of posterior pole remodeling that better reflects its complex spatial nature. Notably, while prior work has emphasized the presence or absence of posterior staphyloma, our findings suggest that meaningful structural variation exists even in eyes without overt staphyloma, supporting the concept of a continuum of posterior pole remodeling^18,24^.

An important and clinically relevant contribution of this study is the characterization of eyes with discordant axial length and refractive phenotypes. Eyes meeting refractive but not axial criteria for high myopia (SER–only high myopia) demonstrated posterior pole morphology that more closely resembled concordant high myopia eyes than concordant non–high myopia eyes. This observation indicates that posterior pole morphology captures structural information that is not fully explained by axial length or refractive status alone^19,20,25^. Such structural–refractive dissociation may help explain why eyes with similar refractive error can exhibit markedly different risks of myopic complications. Our findings suggest that posterior pole morphology may provide additional structural information beyond axial length or refractive status alone^21,24,26^. However, these approaches have been limited by restricted spatial coverage, high cost, or reliance on qualitative classification. Elevation-based mapping approaches were not included in the present analysis, as they require additional reference-surface assumptions and were beyond the scope of the current curvature-based framework. Our study differs in several important aspects.

Quadrant–based analyses further revealed that posterior pole remodeling in high myopia is not uniformly distributed across directions. Both slope–based and expansion–based indices demonstrated asymmetric involvement of specific quadrants, with consistent patterns across metrics. These findings are broadly consistent with previous clinical observations that posterior pole changes in myopia may show regional predilection^22,23^. By quantifying these directional differences, our approach provides an objective framework to study regional vulnerability of the posterior pole and may facilitate future investigations into regional patterns of posterior pole variation.

Although this study was not designed to establish diagnostic thresholds or predictive models, the presented framework offers a clinically interpretable method to complement conventional axial length and refractive assessments. The conceptual decision–support schematic illustrates how posterior pole morphology may add contextual information, particularly in eyes with discordant structural and refractive characteristics. From an ophthalmic perspective, this approach may provide complementary structural information in eyes with discordant axial and refractive characteristics, where conventional measures may not fully capture posterior pole variation. Importantly, widefield OCT–based curvature analysis is noninvasive, widely available, and more accessible than magnetic resonance imaging, making it a practical tool for both research and potential clinical applications^18^. Our repeatability analysis further supports the feasibility of incorporating these metrics into longitudinal studies^17,21^.

The strengths of this study include the use of multiple complementary curvature metrics, rigorous statistical modeling accounting for inter–eye correlation, extensive sensitivity analyses, and cross–center validation. In addition, high repeatability of key indices supports the robustness of the proposed framework. Several limitations should be acknowledged. First, the cross-sectional design precludes inference regarding temporal progression of posterior pole remodeling. Longitudinal studies are needed to determine whether curvature heterogeneity and asymmetry are associated with future myopic complications. Second, the study population consisted primarily of adults from two centers, which may limit generalizability to pediatric populations or other ethnic and clinical settings. Third, although widefield OCT provides broad posterior pole coverage, extremely peripheral regions may still be underrepresented in some eyes with severe staphyloma or marked deformation. Fourth, OCT–based curvature estimation relies on device–specific geometric assumptions and a simplified ocular model; because highly myopic eyes may deviate substantially from this reference geometry, the accuracy of curvature estimates may vary across eyes. Finally, although we provide interpretable curvature–derived indices, these metrics should currently be considered descriptive structural markers rather than diagnostic thresholds or risk predictors.

In summary, this study suggests that posterior pole deformation in high myopia is associated with greater curvature heterogeneity and more pronounced nasal–temporal asymmetry, rather than a uniformly distributed pattern. By quantifying these features using widefield OCT-based curvature analysis, we provide a reproducible and clinically interpretable framework that complements conventional axial length and refractive assessments. These findings suggest that posterior pole morphology may represent an additional structural dimension of high myopia and support further longitudinal and translational investigation.

## Electronic Supplementary Material

Below is the link to the electronic supplementary material.

Supplementary Material 1

Supplementary Material 2

## Data Availability

The data that support the findings of this study are available from the corresponding author upon reasonable request.

## Abbreviations

ACD: Anterior chamber depth
AL: Axial length
BCVA: Best–corrected visual acuity
CCT: Central corneal thickness
dPEI: directional posterior expansion index
FDR: False discovery rate
GEE: Generalized estimating equations
HM: High myopia
ICC: Intraclass correlation coefficient
IOP: Intraocular pressure
Km: Mean keratometry
LMM: Linear mixed–effects model
MRI: Magnetic resonance imaging
non–HM: Non–high myopia
NT: Nasal–temporal
NT diff: Nasal–temporal difference
NT diff slope: Nasal–temporal difference in posterior pole curvature slope (PPslope T − PPslope N)
OCT: Optical coherence tomography
OCTA: Optical coherence tomography angiography
PEI: Posterior expansion index
PHI: Posterior heterogeneity index
PHI slope: Posterior heterogeneity index of curvature slope (SD of PPslope across S/I/N/T)
PPslope: Posterior pole curvature slope
RC: Retinal curvature
R1–R6: Concentric retinal rings from macula (inner) to posterior pole periphery (outer)
SER: Spherical equivalent refraction
SI: Superior–inferior
SS–OCT: Swept–source optical coherence tomography
VCSEL: Vertical–cavity surface–emitting laser
VT: Vitreous thickness
WTW: White–to–white corneal diameter
