# Supplementary material for: Asymmetry of posterior pole remodeling in high myopia
Source: Sci Rep. 2026 Apr 21;16:18586. doi: 10.1038/s41598-026-49683-w (PMC13269795; doi:10.1038/s41598-026-49683-w)
Supplement: Supplementary file 1 — Supplementary Material 1 [file 41598_2026_49683_MOESM1_ESM.docx]

Table of Contents

[Supplementary Methods — Quantification of Posterior Pole Curvature and Directional Expansion Indices 4](#_Toc21280)

[1. Ring-based curvature definitions 4](#_Toc29346)

[2. Posterior Expansion Index (PEI) 4](#_Toc7531)

[3. Quadrant-specific local expansion indices (dPEI) 5](#_Toc2487)

[4. Posterior Pole Curvature Slope (PPslope) 5](#_Toc23947)

[5. Directional bias indices 6](#_Toc15318)

[6. Peripheral Heterogeneity Index (PHI) 6](#_Toc26577)

[Supplementary Figure S1. Representative multimodal visualization of posterior pole morphology across refractive severity. 8](#_Toc21185)

[Supplementary Section A — Repeatability and Reliability of Posterior Pole Morphology Metrics 9](#_Toc26141)

[Supplementary Table S1. Repeatability of posterior pole morphology metrics based on repeated measurements 9](#_Toc26762)

[Supplementary Section B — Comprehensive models & multiplicity 12](#_Toc13202)

[Supplementary Table S2. All curvature-related outcomes (LMM) with BH-FDR. 12](#_Toc3774)

[Supplementary Section C — Center differences and generalizability 16](#_Toc27298)

[Supplementary Table S3. Baseline characteristics of participants by study center. 16](#_Toc26669)

[Supplementary Figure S2. Adjusted mean posterior pole metrics by high myopia status within each center and Center×HM interaction. 17](#_Toc28561)

[Supplementary Figure S3. Center-standardized (within-center z-score) comparison of posterior pole metrics by high myopia status. 18](#_Toc32328)

[Supplementary Section D — Dependency between fellow eyes and alternative modeling strategies 20](#_Toc13484)

[Supplementary Figure S4. One-eye sensitivity analyses using the right eye or a randomly selected eye per participant. 20](#_Toc13912)

[Supplementary Figure S5. Generalized estimating equation (GEE) analyses as an alternative to linear mixed-effects models. 22](#_Toc7550)

[Supplementary Figure S6. Intraclass correlation coefficients (ICC) quantifying inter-eye dependency for posterior pole metrics. 23](#_Toc21369)

[Supplementary Section E — Model Assumptions, Robustness, and Sensitivity Analyses 24](#_Toc8046)

[Supplementary Figure S7. Model diagnostics for linear mixed-effects models (LMMs) Residual 24](#_Toc8527)

[Supplementary Figure S8. Robustness of PHI slope and NT diff slope after trimming extreme values 25](#_Toc7194)

[Supplementary Figure S9. Robustness of PHI slope and NT diff slope after winsorization 26](#_Toc21855)

[Supplementary Figure S10. Sensitivity analyses using alternative definitions of high myopia 27](#_Toc27600)

[Supplementary Section F — Posterior Pole Morphology Patterns and Structural–Refractive Dissociation 28](#_Toc25652)

[Supplementary Figure S11. Ring-based posterior curvature profiles across concentric retinal zones (RC2–RC6) 28](#_Toc148)

[Supplementary Figure S12. Quadrant pattern of directional posterior expansion (dPEI) by high myopia status. 29](#_Toc12477)

[Supplementary Figure S13. Clinically interpretable effect sizes for posterior pole morphology (per +1 mm axial length or per −3 D spherical equivalent) 30](#_Toc3733)

[Supplementary Figure S14. Posterior pole morphology across axial length tertiles within high myopia 30](#_Toc28549)

[Supplementary Figure S15. Posterior pole morphology across spherical equivalent tertiles within high myopia 31](#_Toc9392)

[Supplementary Section G — Discordant AL–SER Phenotypes: Structural–Refractive Dissociation as a Key Exploratory Clinical Scenario 33](#_Toc6886)

[Supplementary Figure S16. Discordant AL–SER phenotype groups: distribution of PHI slope 33](#_Toc27386)

[Supplementary Figure S17. Discordant AL–SER phenotype groups: distribution of NT difference slope 34](#_Toc25234)

[Supplementary Figure S18. Discordant AL–SER phenotype groups: selected supportive posterior pole metrics 35](#_Toc20603)

[Supplementary Table S4. Linear mixed-effects models comparing posterior pole morphology across discordant AL–SER phenotype groups 36](#_Toc16064)

[Panel A. Descriptive summary by phenotype group (eye-level) 36](#_Toc9503)

[Panel B. LMM-adjusted group contrasts (reference = Concordant non-HM) 36](#_Toc10876)

[Supplementary Figure S19. Axial length–spherical equivalent scatter with continuous PHI slope overlay 37](#_Toc8043)

[Supplementary Figure S20. Axial length–spherical equivalent scatter with continuous NT difference slope overlay 38](#_Toc26173)

[Supplementary Figure S21. Axial length–spherical equivalent scatter stratified by binary PHI risk (print-friendly) 39](#_Toc17110)

[Supplementary Figure S22. Planned contrasts focusing on SER-only high myopia phenotype 40](#_Toc29705)

[Supplementary Section H — Exploratory analyses: SER explained by posterior pole morphology beyond axial length 41](#_Toc15593)

[Supplementary Table S5. Incremental explanatory value of posterior pole morphology for spherical equivalent refraction beyond axial length 41](#_Toc30868)

[Panel A. Model fit indices (same sample across models) 41](#_Toc16290)

[Panel B. Key fixed-effect coefficients 42](#_Toc1397)

[Panel C. Grouped 5-fold cross-validation (subject-level grouped CV) 42](#_Toc1906)

[Supplementary Table S6. Continuous linear mixed-effects models using spherical equivalent refraction as the exposure variable 43](#_Toc22988)

[Supplementary Section I — Conceptual schematic 45](#_Toc8634)

[Supplementary Figure S23. Conceptual decision-support schematic illustrating how posterior pole morphology complements conventional axial length (AL) and spherical equivalent refraction (SER) assessment. 45](#_Toc29995)

# ****Supplementary Methods — Quantification of Posterior Pole Curvature and Directional Expansion Indices****

## Posterior pole morphology was quantified using ring-based retinal curvature measurements derived from widefield OCT. Curvature metrics were summarized radially (inner-to-outer rings) and by quadrant (superior, inferior, nasal, and temporal) to characterize global expansion, local asymmetry, and peripheral heterogeneity of the posterior pole. All indices were calculated at the eye level. A schematic overview of the ring- and quadrant-based spatial framework is provided in Figure 1 and Supplementary Figure S1.

## **1. Ring-based curvature definitions**

Retinal curvature was measured within six concentric rings centered on the fovea.

R1 corresponds to the macular (central) region.

R6 corresponds to the outer posterior pole periphery.

Retinal curvature was measured within six concentric rings centered on the fovea, with outer diameters of 1, 3, 6, 9, 12, and 15 mm.

For consistency with prior literature and to enhance robustness against central noise, R2 was used as the inner reference ring in expansion-based indices.

**2. Posterior Expansion Index (PEI)**

The Posterior Expansion Index (PEI) represents the overall radial expansion from the macular-adjacent region to the posterior pole periphery.

$$PEI=-\frac{RC6-RC2}{4}$$

where:

R2 and R6 denote mean retinal curvature values at rings R2 and R6, respectively.

The denominator reflects the number of radial steps between R2 and R6.

The negative sign ensures that larger positive PEI values correspond to greater posterior pole bulging.

**3. Quadrant-specific local expansion indices (dPEI)**

To capture directional variation in posterior expansion, quadrant-specific differential expansion indices were calculated using the same outer–inner ring approach.

$$dPEI S=-(RC S6-RC S2)$$

$$dPEI I=-(RC I6-RC I2)$$

$$dPEI N=-(RC N6-RC N2)$$

$$dPEI T=-(RC T6-RC T2)$$

where subscripts S, I, N, T denote superior, inferior, nasal, and temporal quadrants, respectively. These indices reflect local posterior expansion magnitude along each anatomical direction.

**4. Posterior Pole Curvature Slope (PPslope)**

Posterior pole curvature slope quantifies the rate of curvature change across radial distance, analogous to corneal peripheral steepening.

For each quadrant, PPslope was computed as the slope of a linear regression model:

$$PPslope=\frac{Cov(x,y)}{Var(X)}$$

Where:

x=[2,3,4,5,6] represents ring indices (R2–R6),

y= [RC S2,RC S3, RC S4, RC S5, RC S6] represents corresponding curvature values.

where Cov denotes covariance and Var denotes variance.

Equivalently,

$$PPslope =\frac{Cov(x,y)}{Var(X)}$$

Quadrant-specific slopes were defined as:

PPslope S, PPslope I, PPslope N, PPslope T

Higher slope values indicate steeper posterior pole curvature gradients.

**5. Directional bias indices**

Directional bias indices quantify asymmetry between opposing quadrants, capturing both magnitude and direction of posterior pole remodeling.

Difference-based indices

$$SI diff=dPEI S-dPEI I$$

$$NT diff=dPEI T-dPEI N$$

Slope-based indices

$SI diff slope=PPslope S-PPslope I$

$$NT diff slope=PPslope T-PPslope N$$

Positive values indicate greater expansion or steepening in the first-listed direction (superior or temporal), whereas negative values indicate dominance of the opposing direction.

**6. Peripheral Heterogeneity Index (PHI)**

Peripheral heterogeneity indices quantify directional variability of posterior pole remodeling, independent of absolute expansion magnitude.

Difference-based heterogeneity

$$PHI dPEI=SD(dPEI S,dPEI I,dPEI N,dPEI T)$$

Slope-based heterogeneity

$$PHI\_slope=SD(PPslope S,PPslope I,PPslope N,PPslope T)$$

Larger PHI values indicate greater directional heterogeneity and irregular posterior pole morphology, whereas smaller values suggest a more symmetric and uniform curvature profile.

Abbreviations: AL = Axial length; SER = Spherical equivalent refraction; RC = Retinal curvature; PEI = Posterior Expansion Index; dPEI = Directional Posterior Expansion Index; PPslope = Posterior pole curvature slope; SI = Superior–Inferior; NT = nasal–temporal; PHI = Peripheral Heterogeneity Index; SD = Standard deviation.

## **Supplementary Figure S1. Representative multimodal visualization of posterior pole morphology across refractive severity.**


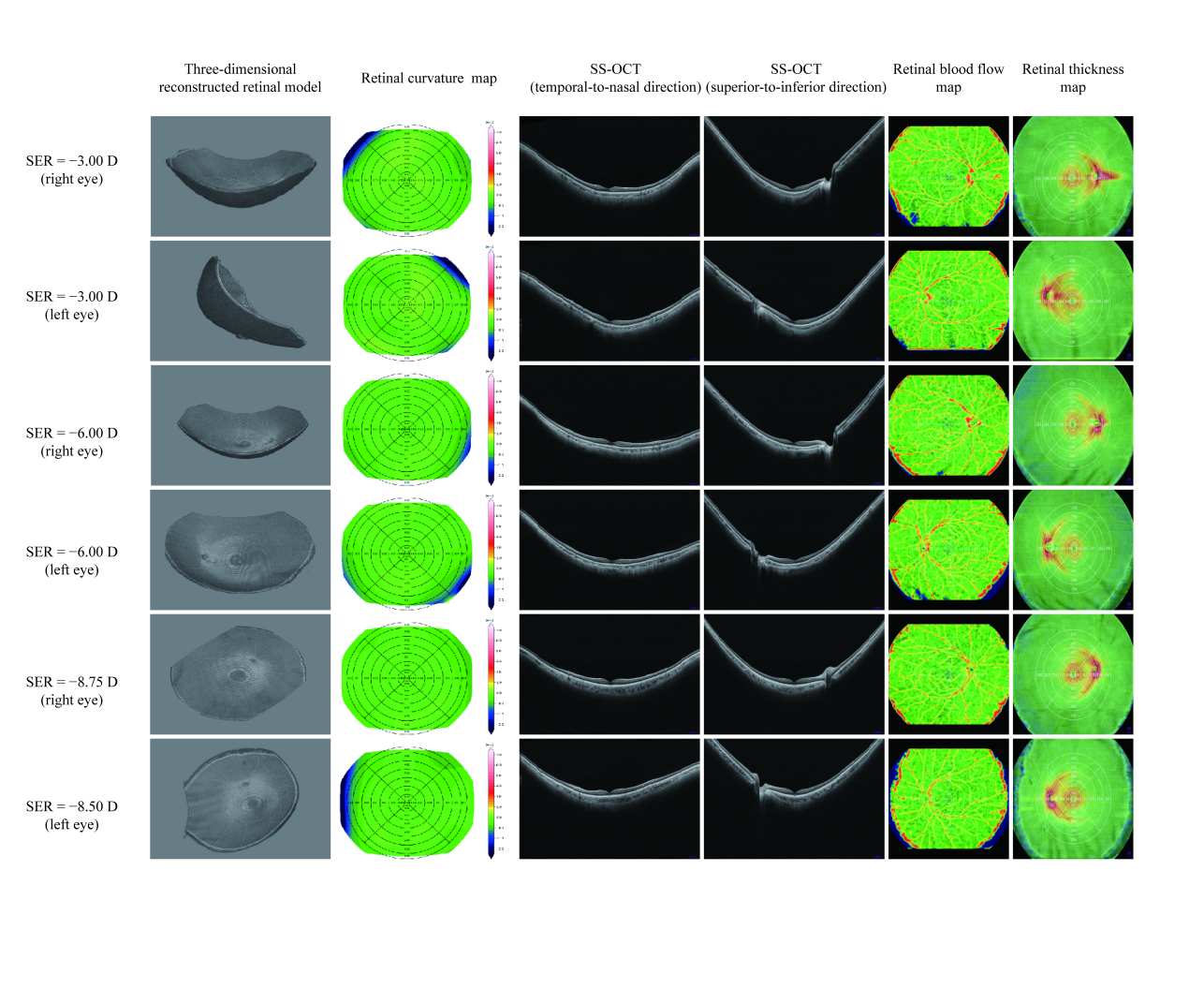

Representative eyes across increasing degrees of myopia are shown to illustrate posterior pole morphological variation. From left to right, panels display: (1) three-dimensional reconstructed retinal surface model; (2) ring-based retinal curvature map centered on the fovea; (3) swept-source optical coherence tomography (SS-OCT) B-scans along the nasal–temporal and superior–inferior axes; (4) OCTA-derived retinal flow-density map; and (5) retinal thickness map. Examples include eyes with mild myopia (spherical equivalent refraction [SER] −3.0 D), high myopia (SER −6.0 D), and severe myopia (SER −8.5 to −8.75 D), shown for both right and left eyes where available. This figure provides qualitative context linking ring- and quadrant-based curvature metrics to three-dimensional posterior pole morphology and multimodal retinal features. These examples are illustrative only and were not used for statistical inference.

### Abbreviations: SER = spherical equivalent refraction; SS-OCT = swept-source optical coherence tomography; D = diopters.

# ****Supplementary Section A — Repeatability and Reliability of Posterior Pole Morphology Metrics****

## To assess the measurement repeatability and reliability of posterior pole morphology metrics, we conducted a dedicated repeated-measures analysis across two independent centers. Both posterior heterogeneity index (PHI slope) and nasal–temporal asymmetry (NT diff slope) were repeatedly measured within and across days to quantify short-term reproducibility. Intraclass correlation coefficients (ICC) were used to evaluate within-center reliability, supporting the robustness of morphology-derived indices used in the main analyses.

**Supplementary Table S1. Repeatability of posterior pole morphology metrics based on repeated measurements**

| **Eye** | **Test 1** | **Test 2** | **Test 3** | **Test 4** | **Mean±SD** |
| --- | --- | --- | --- | --- | --- |
| PHI slope (Center 1) | | | | | |
| Eye1 | 0.048 | 0.047 | 0.046 | 0.045 | 0.046±0.001 |
| Eye2 | 0.061 | 0.06 | 0.055 | 0.061 | 0.059±0.003 |
| Eye3 | 0.012 | 0.016 | 0.01 | 0.014 | 0.013±0.003 |
| Eye4 | 0.014 | 0.02 | 0.022 | 0.019 | 0.019±0.003 |
| Eye5 | 0.019 | 0.024 | 0.024 | 0.022 | 0.022±0.002 |
| Eye6 | 0.025 | 0.022 | 0.02 | 0.022 | 0.022±0.002 |
| Eye7 | 0.028 | 0.023 | 0.026 | 0.025 | 0.026±0.002 |
| Eye8 | 0.014 | 0.012 | 0.015 | 0.021 | 0.015±0.004 |
| Eye9 | 0.068 | 0.064 | 0.066 | 0.073 | 0.068±0.004 |
| Eye10 | 0.032 | 0.03 | 0.032 | 0.029 | 0.031±0.002 |
| NT diff slope (Center 1) | | | | | |
| Eye1 | 0.047 | 0.046 | 0.056 | 0.055 | 0.051±0.005 |
| Eye2 | 0.095 | 0.096 | 0.096 | 0.092 | 0.095±0.002 |
| Eye3 | 0.021 | 0.021 | 0.017 | 0.019 | 0.019±0.002 |
| Eye4 | -0.003 | 0.003 | 0.001 | 0.004 | 0.001±0.003 |
| Eye5 | 0.041 | 0.042 | 0.043 | 0.041 | 0.042±0.001 |
| Eye6 | 0.035 | 0.041 | 0.033 | 0.03 | 0.035±0.005 |
| Eye7 | 0.038 | 0.033 | 0.037 | 0.028 | 0.034±0.005 |
| Eye8 | 0.017 | 0.025 | 0.021 | 0.017 | 0.020±0.004 |
| Eye9 | 0.073 | 0.071 | 0.07 | 0.078 | 0.073±0.004 |
| Eye10 | 0.021 | 0.028 | 0.03 | 0.029 | 0.027±0.004 |
| PHI slope (Center 2) | | | | | |
| Eye1 | 0.054 | 0.053 | 0.051 | 0.051 | 0.052±0.002 |
| Eye2 | 0.034 | 0.034 | 0.027 | 0.035 | 0.033±0.004 |
| Eye3 | 0.046 | 0.052 | 0.043 | 0.049 | 0.048±0.004 |
| Eye4 | 0.12 | 0.128 | 0.132 | 0.127 | 0.127±0.005 |
| Eye5 | 0.059 | 0.066 | 0.066 | 0.064 | 0.064±0.003 |
| Eye6 | 0.042 | 0.037 | 0.035 | 0.038 | 0.038±0.003 |
| Eye7 | 0.073 | 0.067 | 0.071 | 0.069 | 0.070±0.003 |
| Eye8 | 0.093 | 0.091 | 0.094 | 0.103 | 0.095±0.005 |
| Eye9 | 0.03 | 0.024 | 0.027 | 0.037 | 0.029±0.006 |
| Eye10 | 0.077 | 0.074 | 0.076 | 0.072 | 0.075±0.002 |
| NT diff slope (Center 2) | | | | | |
| Eye1 | 0.06 | 0.056 | 0.104 | 0.1 | 0.080±0.026 |
| Eye2 | 0.035 | 0.04 | 0.041 | 0.025 | 0.035±0.007 |
| Eye3 | 0.073 | 0.072 | 0.054 | 0.063 | 0.066±0.009 |
| Eye4 | -0.316 | -0.285 | -0.296 | -0.283 | -0.295±0.015 |
| Eye5 | 0.055 | 0.059 | 0.064 | 0.055 | 0.058±0.004 |
| Eye6 | 0.013 | 0.041 | 0.006 | -0.009 | 0.013±0.021 |
| Eye7 | -0.121 | -0.144 | -0.128 | -0.171 | -0.141±0.022 |
| Eye8 | -0.254 | -0.215 | -0.234 | -0.255 | -0.239±0.019 |
| Eye9 | 0.023 | 0.014 | 0.01 | 0.048 | 0.024±0.017 |
| Eye10 | 0.061 | 0.091 | 0.105 | 0.098 | 0.089±0.019 |

Posterior pole morphology metrics were measured four times per eye, including three repeated scans on Day 1 and one additional scan on Day 2. Values are presented at the eye level. Mean ± SD summarizes within-eye variability across repeated measurements. Measurements were conducted independently at two clinical centers using identical acquisition and processing protocols.

Abbreviations: PHI slope = Posterior heterogeneity index slope; NT diff slope = nasal–temporal curvature difference slope; ICC = Intraclass correlation coefficient

; SD = Standard deviation.

**Supplementary Section B — Comprehensive models & multiplicity**

This section provides expanded analyses across the full set of posterior pole curvature–related outcomes derived from OCTA 24×20 scans. To ensure robustness of inference given the number of correlated structural metrics, linear mixed-effects models were applied consistently and results were corrected for multiple comparisons using the Benjamini–Hochberg false discovery rate procedure. These analyses complement the primary outcomes by characterizing the broader structural phenotype associated with high myopia.

**Supplementary Table S2. All curvature-related outcomes (LMM) with BH-FDR.**

| **index** | **Total eyes** | **β (95% CI)** | ***P* value** | **FDR *P* value** |
| --- | --- | --- | --- | --- |
| RC T6 | 286 | -0.158 [-0.200, -0.115] | **<0.001** | **<0.001** |
| RC 6 | 286 | -0.357 [-0.473, -0.241] | **<0.001** | **<0.001** |
| RC S II | 286 | -0.271 [-0.364, -0.177] | **<0.001** | **<0.001** |
| RC S6 | 286 | -0.114 [-0.154, -0.074] | **<0.001** | **<0.001** |
| NT diff | 286 | 0.208 [0.131, 0.285] | **<0.001** | **<0.001** |
| NT diff slope | 286 | -0.058 [-0.081, -0.036] | **<0.001** | **<0.001** |
| RC S5 | 286 | -0.097 [-0.134, -0.059] | **<0.001** | **<0.001** |
| dPEI T | 286 | 0.189 [0.112, 0.266] | **<0.001** | **<0.001** |
| PPslope T | 286 | -0.051 [-0.072, -0.030] | **<0.001** | **<0.001** |
| RC S | 286 | -0.323 [-0.460, -0.186] | **<0.001** | **<0.001** |
| RC T II | 286 | -0.201 [-0.295, -0.107] | **<0.001** | **<0.001** |
| RC T5 | 286 | -0.071 [-0.108, -0.034] | **<0.001** | **<0.001** |
| RC Peripheral | 286 | -0.526 [-0.816, -0.235] | **<0.001** | **0.002** |
| RC S4 | 286 | -0.068 [-0.107, -0.030] | **<0.001** | **0.002** |
| RC I6 | 286 | -0.073 [-0.114, -0.031] | **<0.001** | **0.003** |
| PEI | 286 | 0.093 [0.036, 0.149] | **0.001** | **0.005** |
| PHI slope | 286 | 0.012 [0.004, 0.020] | **0.005** | **0.015** |
| PHI dPEI | 286 | 0.040 [0.012, 0.068] | **0.005** | **0.016** |
| RC N3 | 286 | -0.054 [-0.093, -0.016] | **0.006** | **0.018** |
| dPEI S | 286 | 0.094 [0.026, 0.162] | **0.007** | **0.019** |
| RC 5 | 286 | -0.157 [-0.272, -0.043] | **0.007** | **0.019** |
| PPslope S | 286 | -0.024 [-0.043, -0.006] | **0.008** | **0.021** |
| dPEI I | 286 | 0.086 [0.022, 0.150] | **0.009** | **0.022** |
| RC N I | 286 | -0.089 [-0.165, -0.014] | **0.021** | **0.047** |
| PPslope I | 286 | -0.020 [-0.037, -0.003] | **0.020** | **0.047** |
| RC T3 | 286 | 0.063 [0.006, 0.119] | **0.029** | 0.062 |
| RC S3 | 286 | -0.039 [-0.081, 0.002] | 0.062 | 0.129 |
| RC Total | 286 | -0.480 [-0.999, 0.039] | 0.069 | 0.139 |
| RC T I | 286 | 0.095 [-0.012, 0.202] | 0.081 | 0.156 |
| RC N2 | 286 | -0.034 [-0.074, 0.006] | 0.092 | 0.171 |
| RC I4 | 286 | 0.035 [-0.008, 0.079] | 0.111 | 0.194 |
| RC N4 | 286 | -0.036 [-0.080, 0.008] | 0.108 | 0.194 |
| RC N | 286 | -0.125 [-0.282, 0.031] | 0.115 | 0.195 |
| RC S I | 286 | -0.056 [-0.139, 0.027] | 0.184 | 0.294 |
| RC T4 | 286 | 0.033 [-0.016, 0.083] | 0.181 | 0.294 |
| RC T | 286 | -0.098 [-0.259, 0.063] | 0.233 | 0.344 |
| RC T2 | 286 | 0.033 [-0.020, 0.085] | 0.221 | 0.344 |
| SI diff slope | 286 | -0.009 [-0.025, 0.006] | 0.233 | 0.344 |
| RC I II | 286 | -0.056 [-0.159, 0.047] | 0.287 | 0.412 |
| SI diff | 286 | 0.028 [-0.026, 0.082] | 0.315 | 0.441 |
| PPslope N | 286 | 0.008 [-0.009, 0.024] | 0.366 | 0.500 |
| RC I3 | 286 | 0.018 [-0.023, 0.059] | 0.381 | 0.508 |
| RC S2 | 286 | -0.018 [-0.061, 0.026] | 0.427 | 0.556 |
| dPEI N | 286 | -0.020 [-0.081, 0.040] | 0.508 | 0.647 |
| RC I I | 286 | 0.026 [-0.057, 0.108] | 0.539 | 0.671 |
| RC N II | 286 | -0.033 [-0.146, 0.079] | 0.561 | 0.683 |
| RC N6 | 286 | -0.011 [-0.053, 0.030] | 0.585 | 0.698 |
| RC I5 | 286 | -0.010 [-0.054, 0.033] | 0.640 | 0.747 |
| RC 3 | 286 | 0.031 [-0.115, 0.178] | 0.676 | 0.773 |
| RC I2 | 286 | 0.008 [-0.036, 0.052] | 0.715 | 0.800 |
| RC I | 286 | -0.015 [-0.165, 0.136] | 0.847 | 0.930 |
| RC Central | 286 | 0.030 [-0.324, 0.384] | 0.867 | 0.934 |
| RC 4 | 286 | 0.006 [-0.127, 0.140] | 0.925 | 0.960 |
| RC N5 | 286 | 0.002 [-0.044, 0.048] | 0.924 | 0.960 |
| RC 2 | 286 | 0.004 [-0.166, 0.174] | 0.960 | 0.978 |
| RC 1 | 286 | -0.000 [-0.045, 0.045] | 0.993 | 0.993 |

Models and outcome definition. For each curvature-derived metric listed, we fitted a linear mixed-effects model at the eye level to compare high myopia (HM) versus non–high myopia (non-HM) while accounting for inter-eye correlation using a subject-specific random intercept. The model was: metric ~ HM + Age + Sex + Eye laterality + Center + (1 | ID). where ID denotes participant identifier (random intercept), and Center denotes study site. HM was defined as axial length (AL) ≥ 26.0 mm or spherical equivalent refraction (SER) ≤ −6.00 D. Reporting. The table reports the fixed-effect coefficient β for HM (HM vs non-HM), its 95% confidence interval (95% CI), the two-sided P value, and the Benjamini–Hochberg false discovery rate–adjusted P value (P FDR) across all listed outcomes. Total eyes indicate the number of participants and eyes included for each model after complete-case inclusion for that outcome. Interpretation. Positive β indicates higher values of the metric in HM compared with non–HM; negative β indicates lower values in HM. Curvature-related outcomes are reported in the study’s native units (10⁻² mm⁻²) as derived from OCTA 24×20 posterior pole analysis.

Abbreviations: ACD = anterior chamber depth; AL = axial length; BH-FDR / FDR = Benjamini–Hochberg false discovery rate; CI = confidence interval; dPEI = quadrant-specific posterior expansion index difference (outer–inner curvature difference); HM = high myopia; ID = participant identifier; I/N/S/T = inferior/nasal/superior/temporal (quadrants); IOP = intraocular pressure; LMM = linear mixed-effects model; NT diff / NT diff slope = nasal–temporal difference (or slope difference) between temporal and nasal metrics; PEI = posterior expansion index; PHI = posterior heterogeneity index (e.g., SD across quadrants); PPslope = posterior pole curvature slope across rings (quadrant-specific); RC = ring curvature (concentric ring zones); SER = spherical equivalent refraction; SI diff / SI diff slope = superior–inferior difference (or slope difference) between superior and inferior metrics; SD = standard deviation.

**Supplementary Section C — Center differences and generalizability**

This section examines potential center-related differences and evaluates the generalizability of the main findings across study sites. Baseline characteristics are compared by center, followed by center-by–high myopia interaction analyses and within-center standardized comparisons. Together, these analyses assess whether the observed associations are consistent across centers rather than driven by site-specific characteristics.

**Supplementary Table S3. Baseline characteristics of participants by study center.**

| **Characteristic** | **Center 1** | **Center 2** | ***P* value** |
| --- | --- | --- | --- |
| Total | 91 | 52 |  |
| High myopia (HM) | 37 (40.7%) | 33 (63.5%) | **0.014** |
| Age (years) | 33.00 [24.50, 39.00] | 35.00 [27.00, 41.25] | 0.466 |
| AL | 25.23 [24.46, 25.99] | 25.98 [24.72, 27.03] | **0.012** |
| SER | -4.88 [-6.56, -2.75] | -6.50 [-8.28, -4.09] | **0.009** |
| IOP | 15.20 [13.12, 17.07] | 14.60 [13.05, 16.48] | 0.176 |
| CCT | 535.00 [514.25, 558.25] | 526.50 [511.50, 546.62] | 0.177 |
| ACD | 3.71 [3.53, 3.90] | 3.66 [3.43, 3.84] | 0.135 |
| AL/CR | 3.25 [3.12, 3.34] | 3.33 [3.18, 3.43] | **0.050** |
| K1 | 43.05 [42.37, 43.94] | 42.74 [42.02, 43.82] | 0.214 |
| K2 | 43.80 [43.13, 44.66] | 43.63 [42.70, 44.46] | 0.296 |
| Km | 43.46 [42.84, 44.17] | 43.29 [42.49, 44.21] | 0.224 |
| WTW | 11.73 [11.50, 11.96] | 11.78 [11.59, 11.95] | 0.664 |
| Sex |  |  | 0.384 |
| Female | 55 (60.4%) | 36 (69.2%) |  |
| Male | 36 (39.6%) | 16 (30.8%) |  |

Baseline characteristics are summarized by study center to describe between-center differences in the enrolled cohort. Continuous variables are presented as **median [Q1, Q3]** or **mean ± SD**, as appropriate, and categorical variables as **n (%)**. P values (two-sided) are provided for descriptive comparison between centers using **Wilcoxon rank-sum tests** for non-normally distributed continuous variables, **Welch two-sample t tests** for approximately normally distributed continuous variables, and **Pearson χ² tests** for categorical variables. This table is intended for **descriptive transparency** and does not affect the prespecified primary analyses, in which **Center** is included as an adjustment covariate.

Abbreviations: AL = axial length; ACD = anterior chamber depth; CCT = central corneal thickness; IOP = intraocular pressure; SER = spherical equivalent refraction; WTW = white-to-white corneal diameter; SD = standard deviation.

**Supplementary Figure S2. Adjusted mean posterior pole metrics by high myopia status within each center and Center×HM interaction.**

**
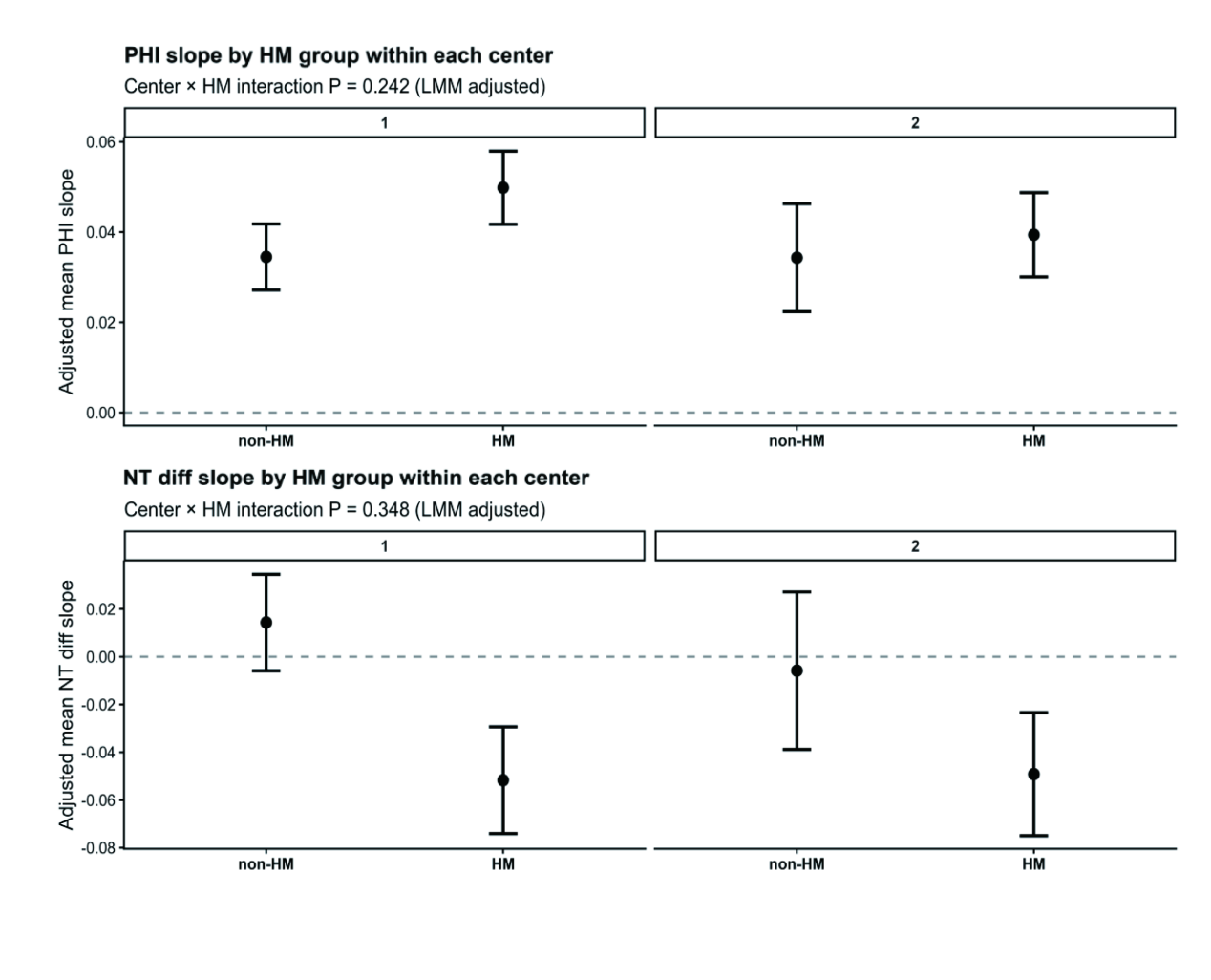
**

This figure evaluates whether the association between high myopia (HM) and posterior pole morphology differs by study center. For each outcome (PHI slope and NT diff slope), we fitted a linear mixed-effects model (LMM) with a Center×HM interaction: Outcome ~ HM × Center + Age + Sex + Eye laterality + (1 | ID). Adjusted means (estimated marginal means) and 95% confidence intervals are displayed for HM and non-HM within each center. The Center×HM interaction P value is reported to assess effect modification by center. Findings are interpreted as evidence of between-center consistency when interaction P values are non-significant and center-specific differences are directionally concordant. This analysis is supportive and complements the primary pooled models.

Abbreviations: HM = high myopia; LMM = linear mixed-effects model; CI = confidence interval; ID = participant identifier; PHI = posterior heterogeneity index; NT diff slope = nasal–temporal difference in posterior pole curvature slope.

**Supplementary Figure S3. Center-standardized (within-center z-score) comparison of posterior pole metrics by high myopia status.**


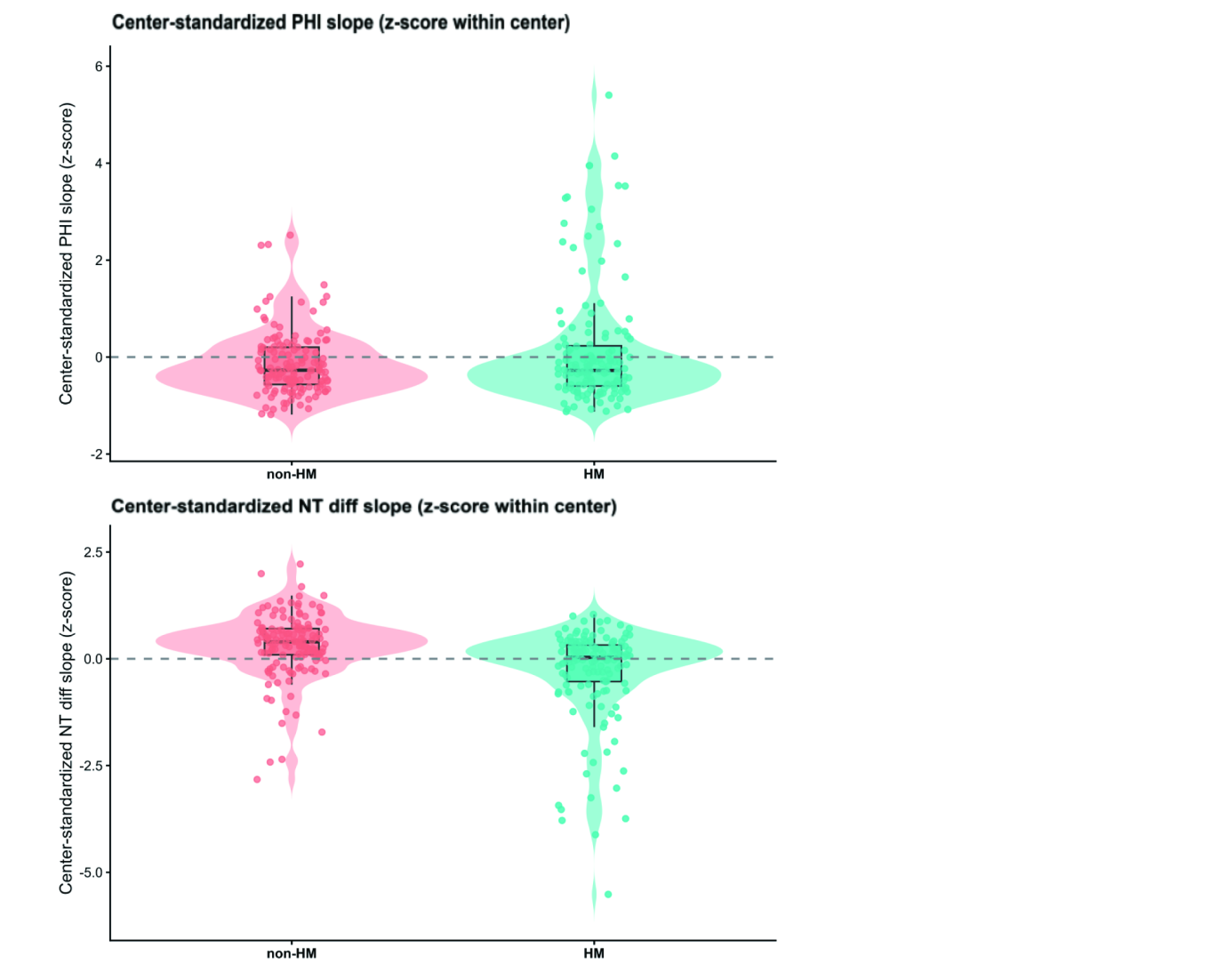


To provide an additional visual check that observed HM vs non–HM differences are not driven by center-specific measurement scale differences, PHI slope and NT diff slope were standardized within each center (z-score transformation: subtract the center mean and divide by the center SD). The distributions of center-standardized outcomes are then compared between HM and non–HM using the same visualization style as in the main figures. This analysis is descriptive and intended to enhance interpretability of cross-center comparability; it does not replace the prespecified primary LMM analyses.

Abbreviations: HM = high myopia; SD = standard deviation; PHI = posterior heterogeneity index; NT diff slope = nasal–temporal difference in posterior pole curvature slope; z-score = standardized score.

**Supplementary Section D — Dependency between fellow eyes and alternative modeling strategies**

Because both eyes from the same participant were included in the primary analyses, this section addresses potential within-subject dependency and evaluates alternative modeling strategies. Sensitivity analyses using single-eye designs, generalized estimating equations, and null mixed models are presented to quantify inter-eye correlation and confirm that the main conclusions are not driven by analytic assumptions. These results support the appropriateness of the primary modeling framework.

**Supplementary Figure S4. One-eye sensitivity analyses using the right eye or a randomly selected eye per participant.**

**
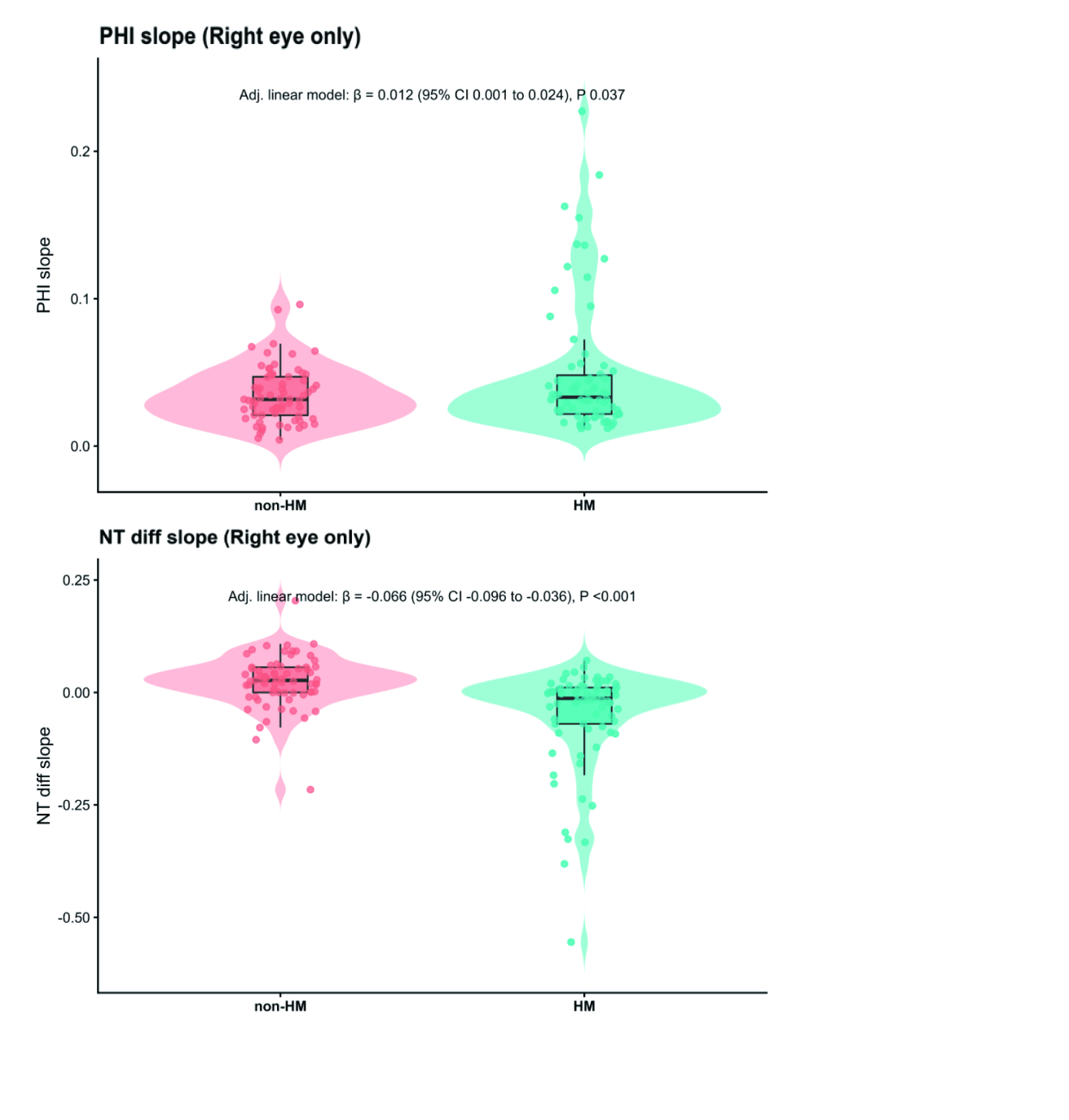
**


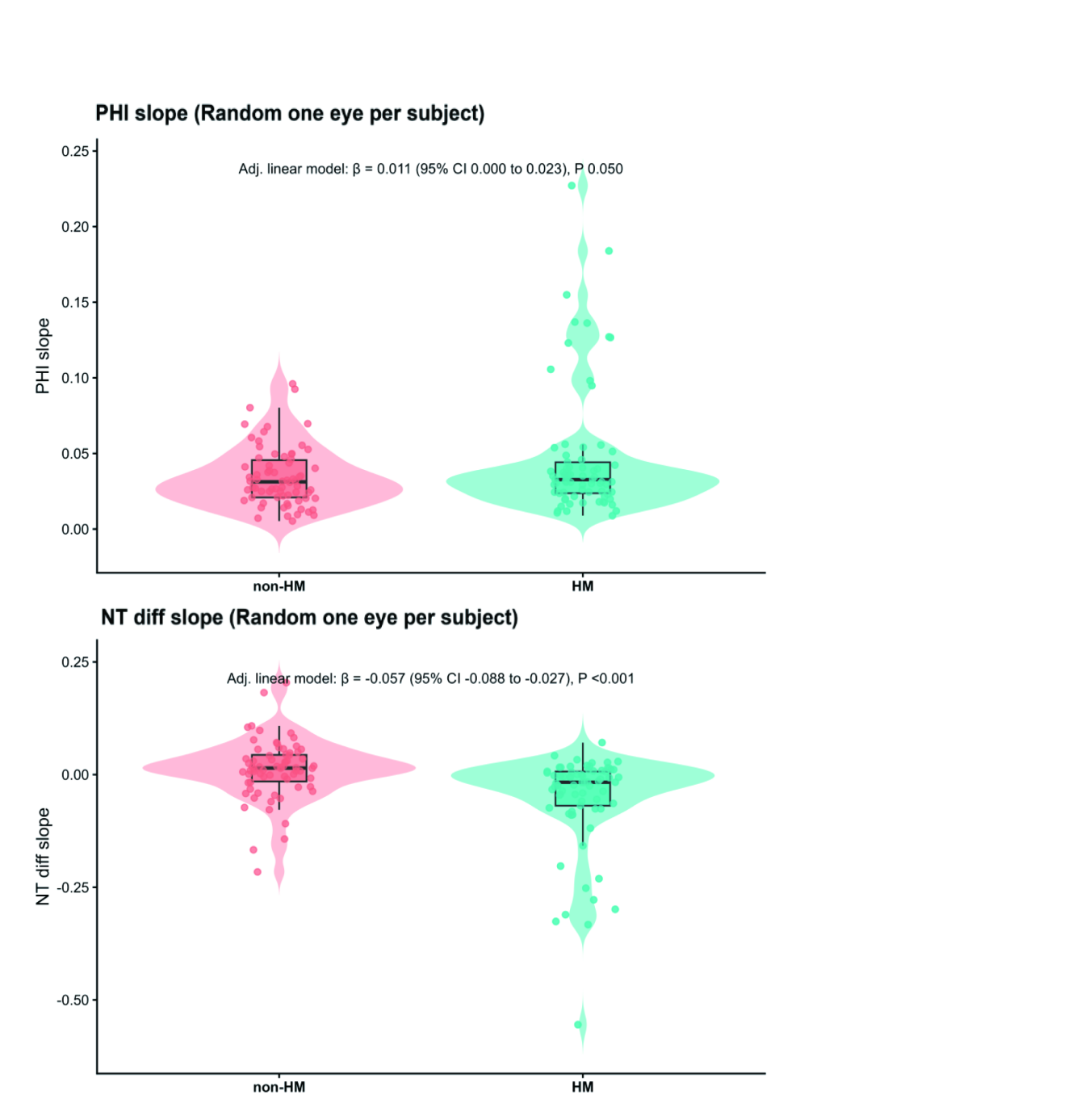


To exclude potential bias arising from inter-eye correlation, the primary analyses were repeated using one eye per participant. Two prespecified approaches were applied: (1) restriction to the right eye for all participants and (2) selection of a single eye at random for each participant. The same covariates and model structure as in the primary analyses were used. Effect estimates and directions for PHI slope and NT diff slope were compared with the main results to assess robustness. Concordant estimates across approaches support the stability of the primary findings.

Abbreviations: PHI = posterior heterogeneity index; NT diff slope = nasal–temporal difference in posterior pole curvature slope.

**Supplementary Figure S5. Generalized estimating equation (GEE) analyses as an alternative to linear mixed-effects models.**

**
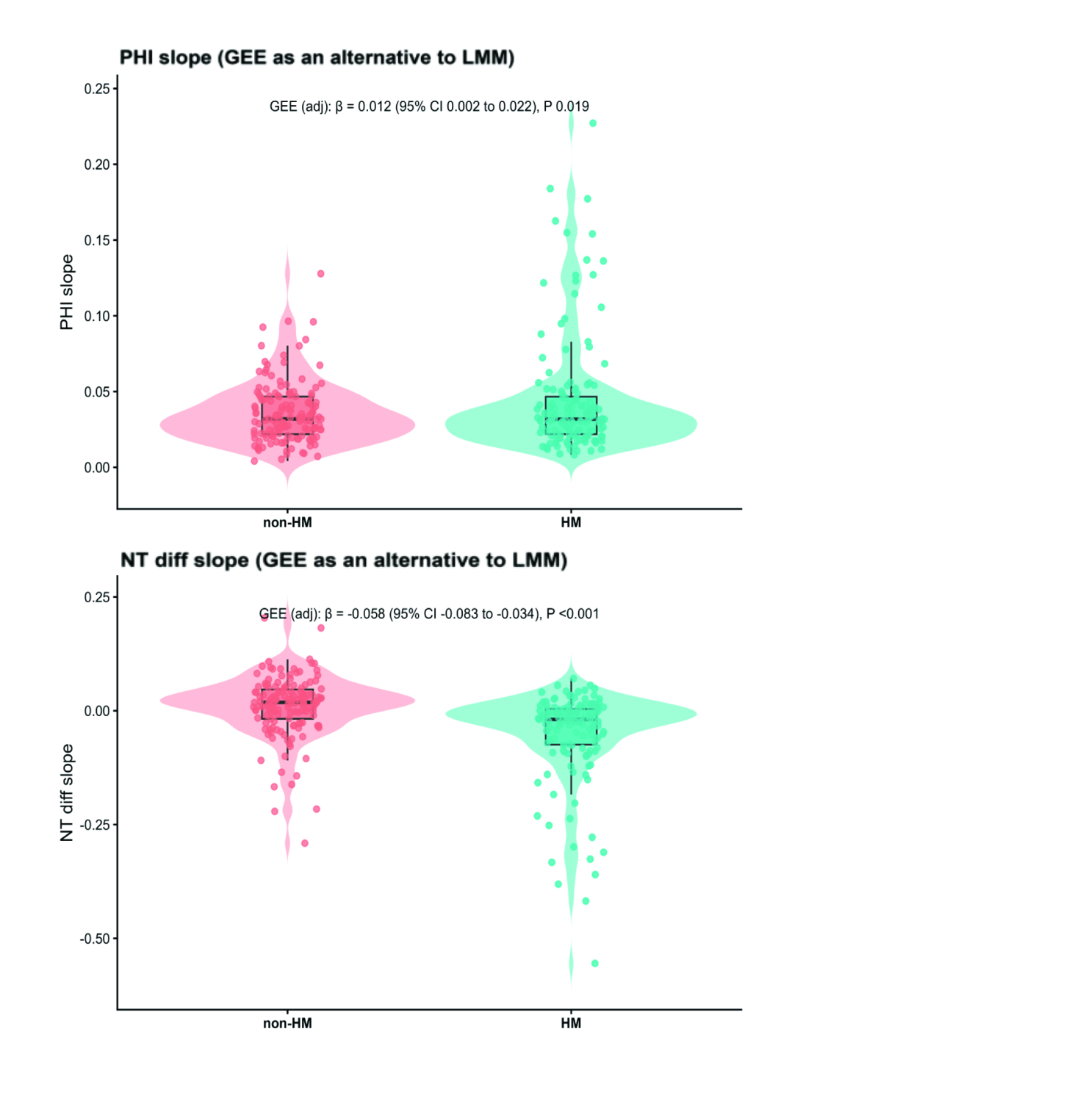
**

To assess whether conclusions were sensitive to the choice of modeling framework for correlated eye-level data, the primary analyses were repeated using generalized estimating equations (GEE) with an exchangeable working correlation structure at the participant level. Results from GEE were compared with those from linear mixed-effects models (LMMs) in terms of effect direction, magnitude, and statistical inference for PHI slope and NT diff slope. Consistency between methods indicates that the primary findings are not driven by the specific modeling approach.

Abbreviations: GEE = generalized estimating equations; LMM = linear mixed-effects model; PHI = posterior heterogeneity index; NT diff slope = nasal–temporal difference in posterior pole curvature slope.

**Supplementary Figure S6. Intraclass correlation coefficients (ICC) quantifying inter-eye dependency for posterior pole metrics.**


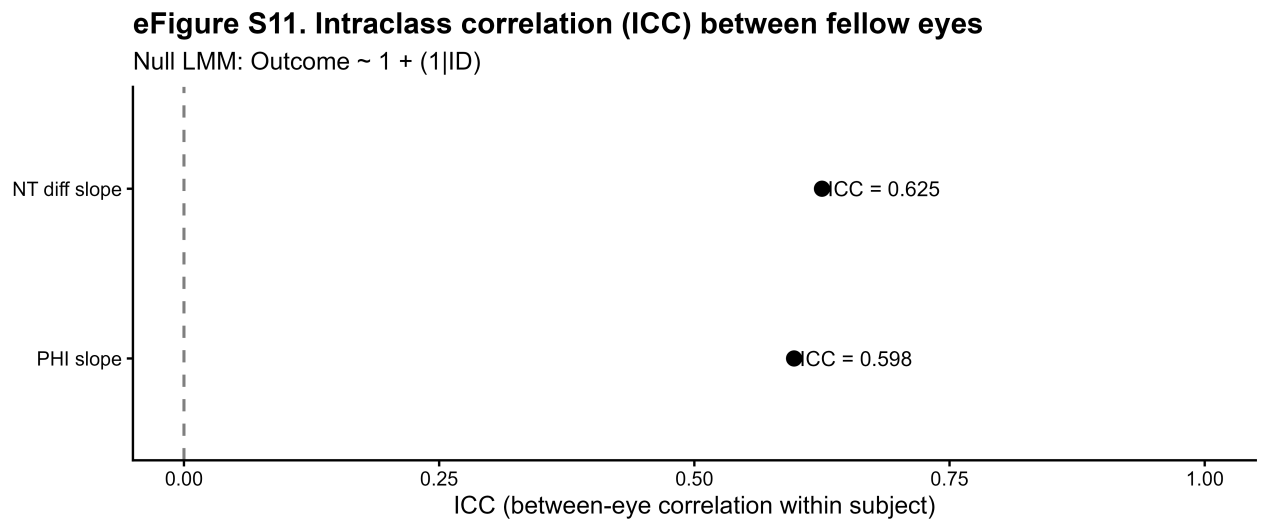


To quantify the degree of correlation between fellow eyes, a null linear mixed-effects model was fitted for each outcome with a random intercept for participant only: outcome ~ 1 + (1 | ID). Intraclass correlation coefficients (ICC) were calculated as the ratio of between-participant variance to total variance. ICC values are reported for PHI slope and NT diff slope, providing a quantitative justification for modeling approaches that account for inter-eye dependency (e.g., LMM or GEE).

Abbreviations: ICC = intraclass correlation coefficient; LMM = linear mixed-effects model; ID = participant identifier; PHI = posterior heterogeneity index; NT diff slope = nasal–temporal difference in posterior pole curvature slope.

**Supplementary Section E — Model Assumptions, Robustness, and Sensitivity Analyses**

This section evaluates key assumptions of the linear mixed-effects models and the robustness of the findings to distributional features of the data. Diagnostic plots, tail-handling strategies, and alternative definitions of high myopia are examined to assess sensitivity to outliers and modeling choices. These analyses demonstrate that the primary results are stable across a range of reasonable analytic variations.

**Supplementary Figure S7. Model diagnostics for linear mixed-effects models (LMMs)**

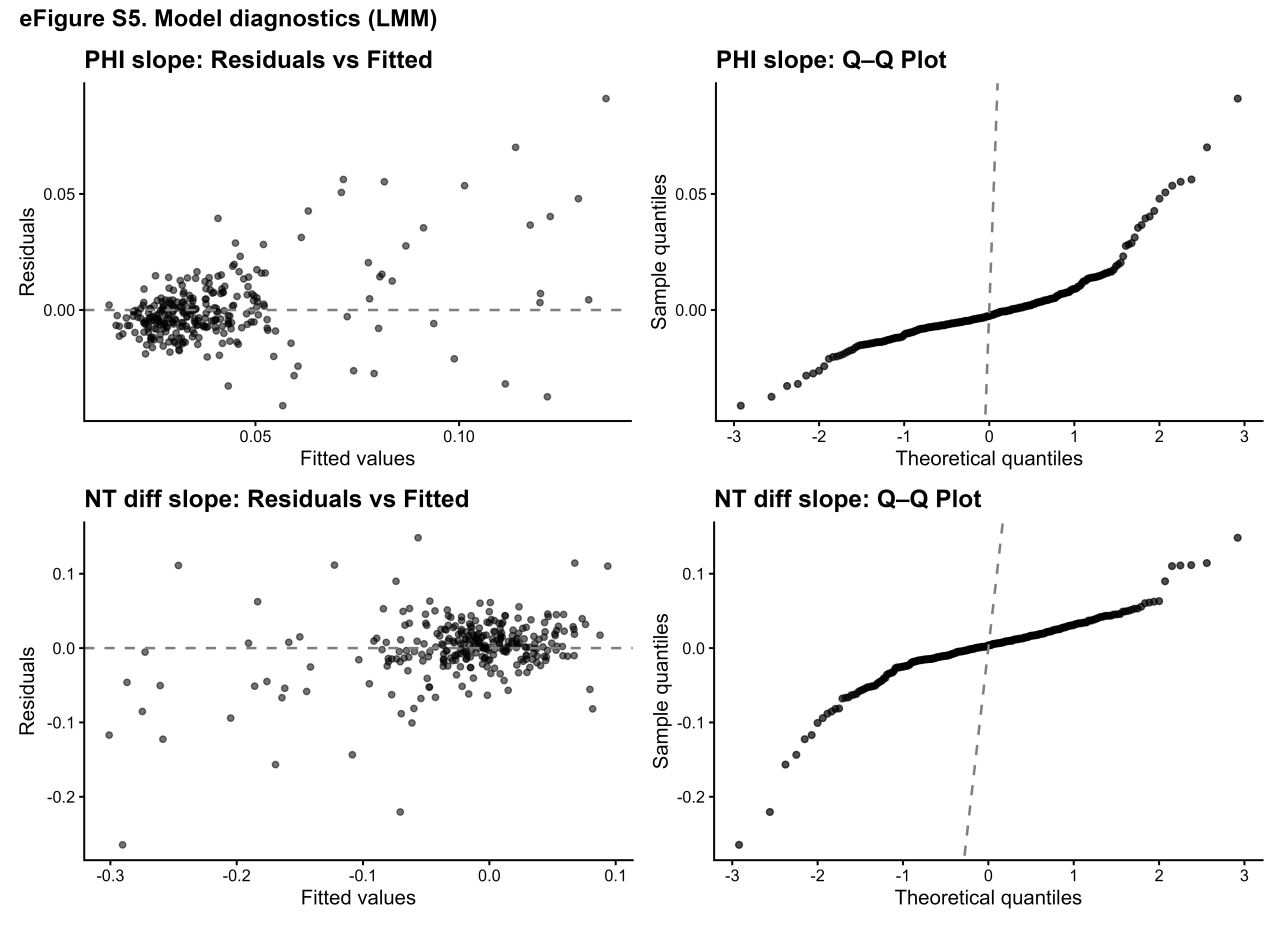
Residual diagnostics for the primary linear mixed-effects models evaluating posterior pole morphology. Shown are residuals versus fitted values and normal Q–Q plots for PHI slope and NT diff slope. Visual inspection demonstrated no substantial deviation from homoscedasticity or normality assumptions, supporting the appropriateness of linear mixed-effects modeling for these outcomes.

Abbreviations: PHI = posterior heterogeneity index; NT = nasal–temporal; LMM = linear mixed-effects model; CI = confidence interval.

**Supplementary Figure S8. Robustness of PHI slope and NT diff slope after trimming extreme values**


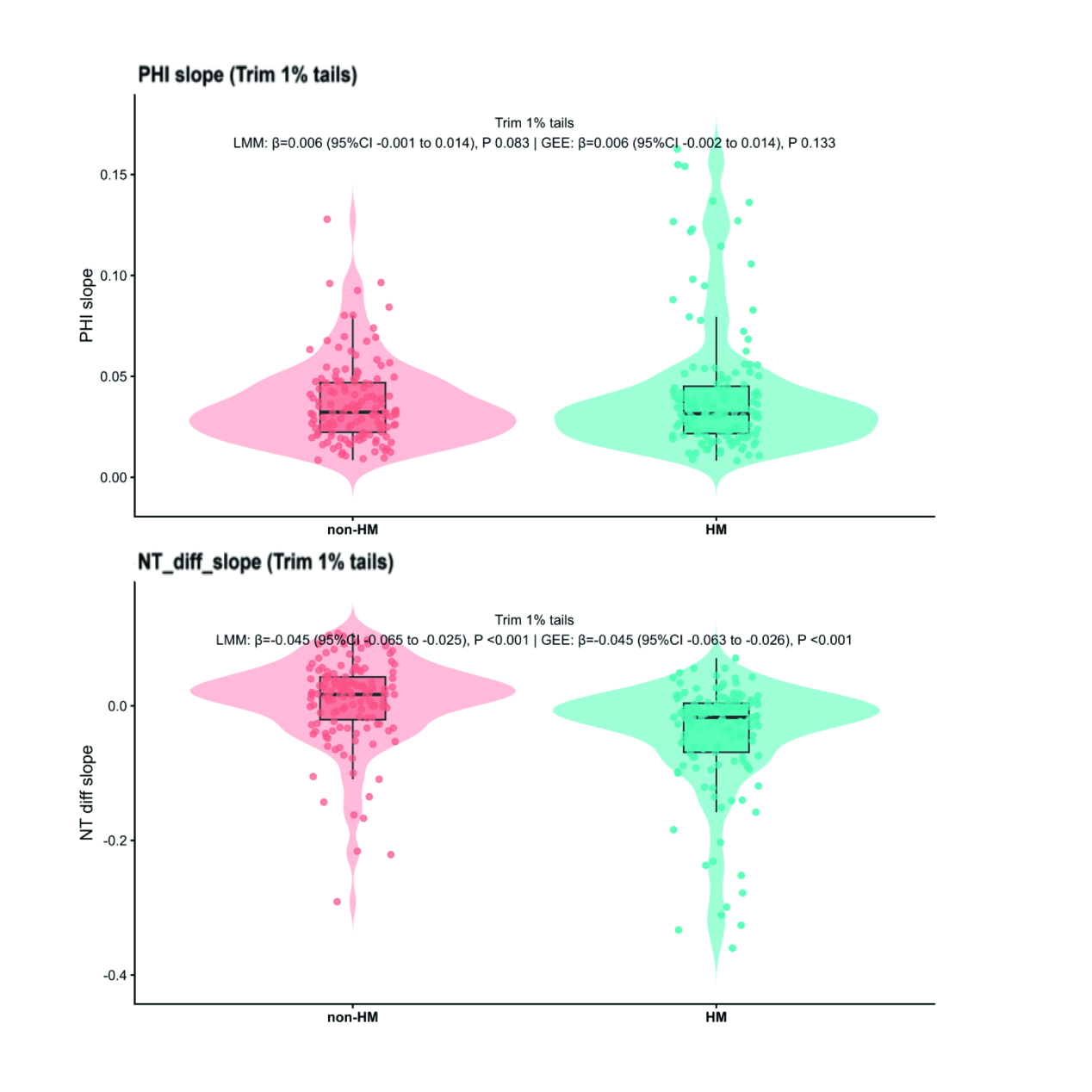


Sensitivity analyses were conducted by trimming the upper and lower 1% tails of PHI slope and NT diff slope distributions. Effect estimates derived from trimmed datasets were compared with the primary analyses to assess robustness to extreme values. Overall patterns and directions of associations remained consistent with the main results.

**Supplementary Figure S9. Robustness of PHI slope and NT diff slope after winsorization**


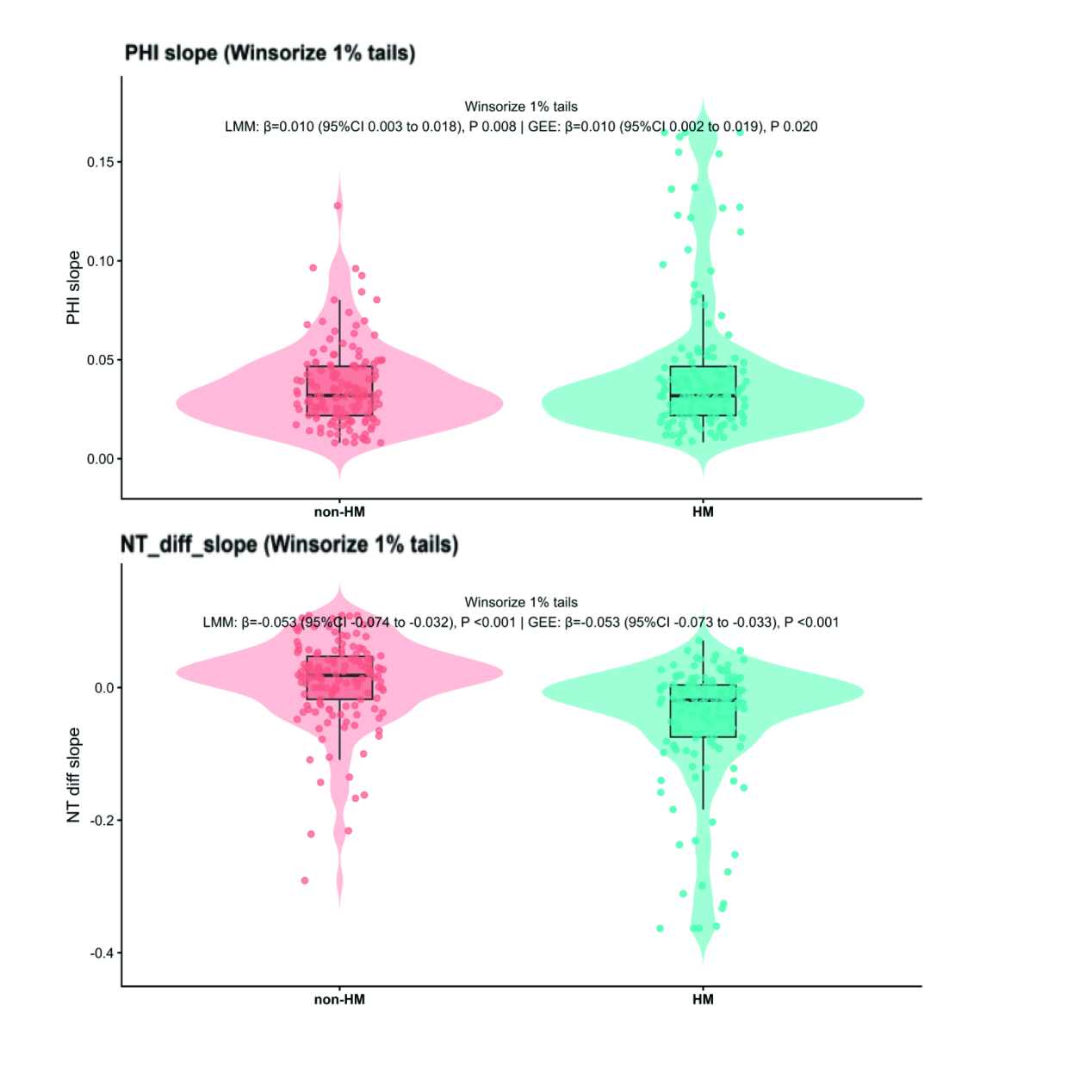

As an alternative approach to handling extreme values, winsorization at the 1% tails was applied to PHI slope and NT diff slope. Results were highly consistent with the primary and trimmed analyses, indicating that findings were not driven by a small number of extreme observations.

**Supplementary Figure S10. Sensitivity analyses using alternative definitions of high myopia**


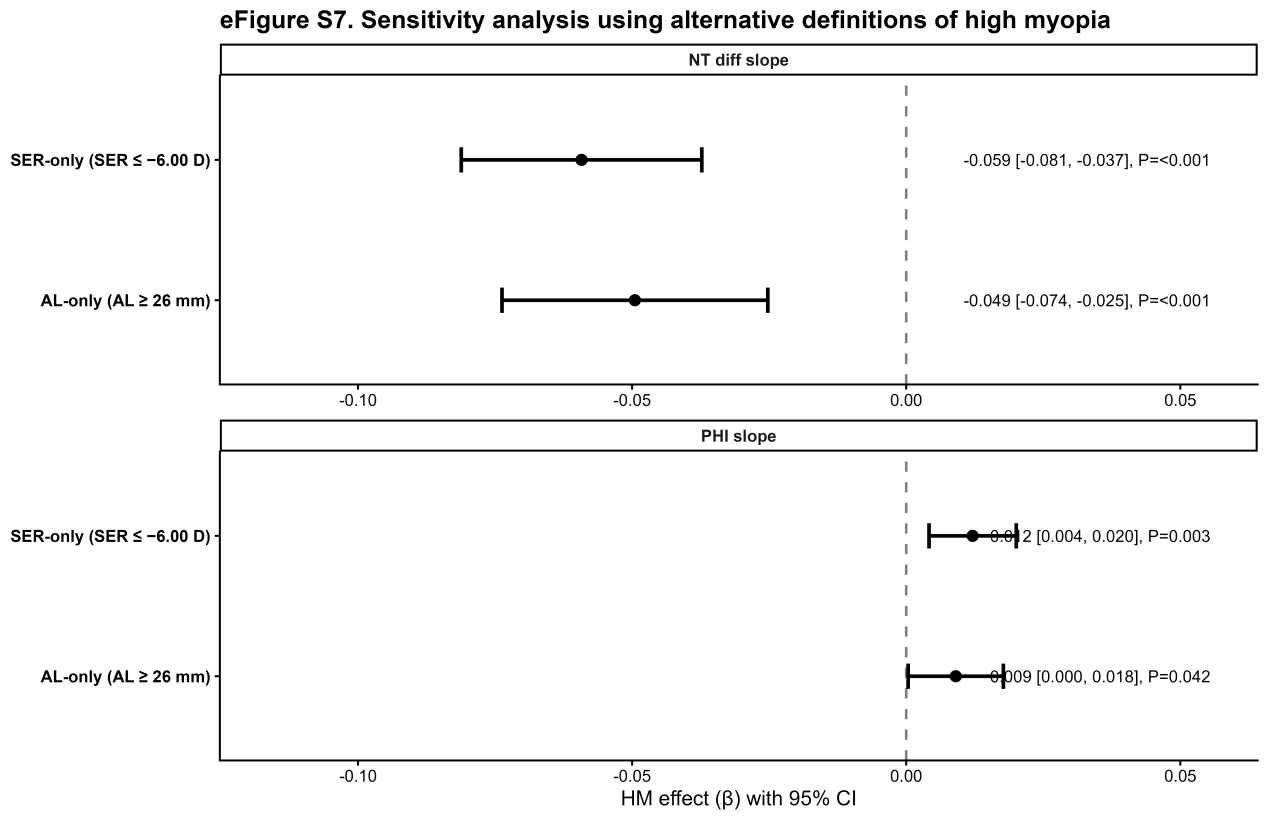

To evaluate the robustness of findings to the operational definition of high myopia (HM), sensitivity analyses were performed using alternative criteria, including axial length–only (AL-only) and spherical equivalent refraction–only (SER-only) definitions. Forest plots display adjusted effect estimates (β) with 95% confidence intervals for PHI slope and NT diff slope. The direction and magnitude of associations were consistent across definitions, supporting the stability of the primary results.

Abbreviations: AL = axial length; SER = spherical equivalent refraction; HM = high myopia; PHI = posterior heterogeneity index; NT = nasal–temporal; LMM = linear mixed-effects model; CI = confidence interval.

**Supplementary Section F — Posterior Pole Morphology Patterns and Structural–Refractive Dissociation**

This section explores spatial and phenotypic patterns of posterior pole morphology and their relationship to refractive status. Ring-based curvature profiles, clinically interpretable effect-size transformations, severity stratification, and analyses of discordant axial length–refraction phenotypes are presented. Collectively, these analyses illustrate how posterior pole morphology provides complementary structural information beyond conventional axial length and refraction measures.

**Supplementary Figure S11. Ring-based posterior curvature profiles across concentric retinal zones (RC2–RC6)**

**
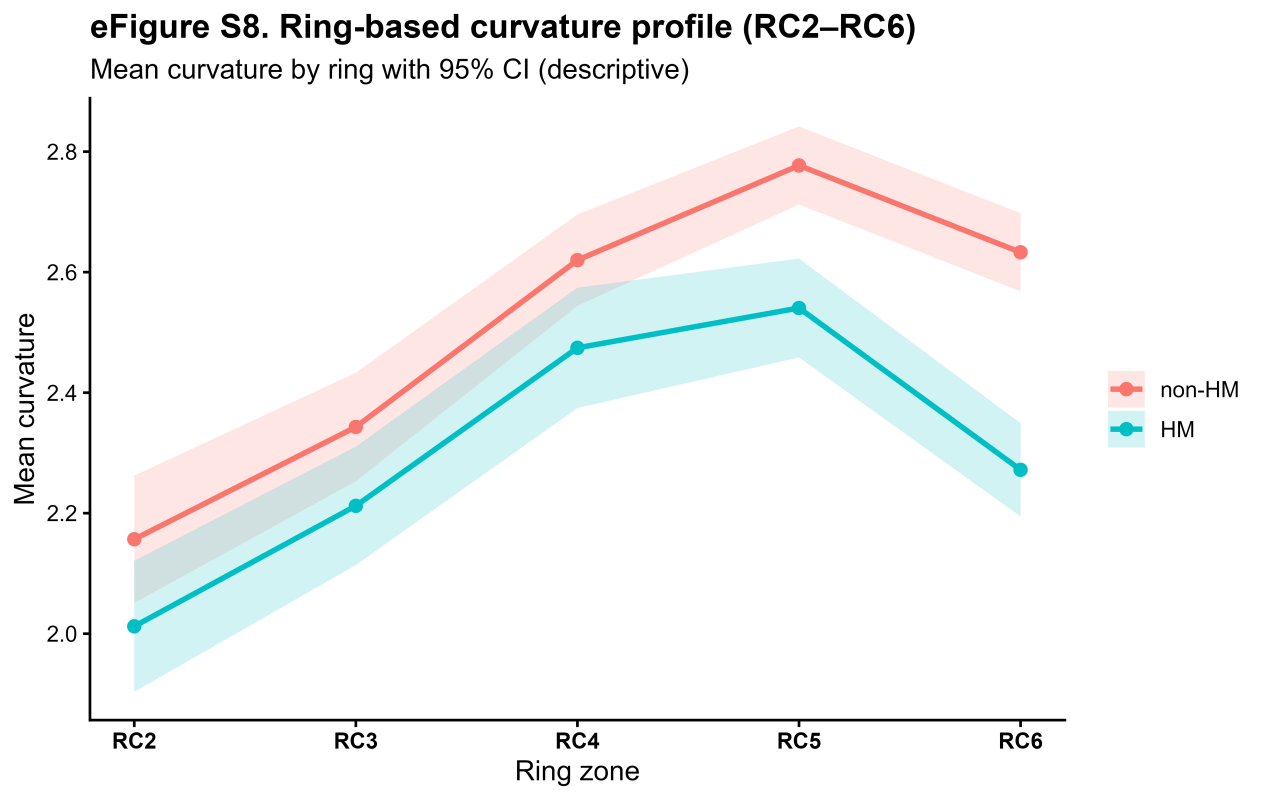
**

Mean posterior curvature values across concentric retinal rings (RC2–RC6) are shown for high myopia (HM) and non-HM eyes. Lines represent group means, with shaded areas indicating 95% confidence intervals. This descriptive analysis illustrates spatial patterns of posterior pole curvature and highlights regional differences across increasing eccentricity from the fovea. The profiles provide anatomical context for subsequent analyses of posterior pole remodeling.

Abbreviations: RC = retinal curvature ring; HM = high myopia; CI = confidence interval.

**Supplementary Figure S12. Quadrant pattern of directional posterior expansion (dPEI) by high myopia status.**

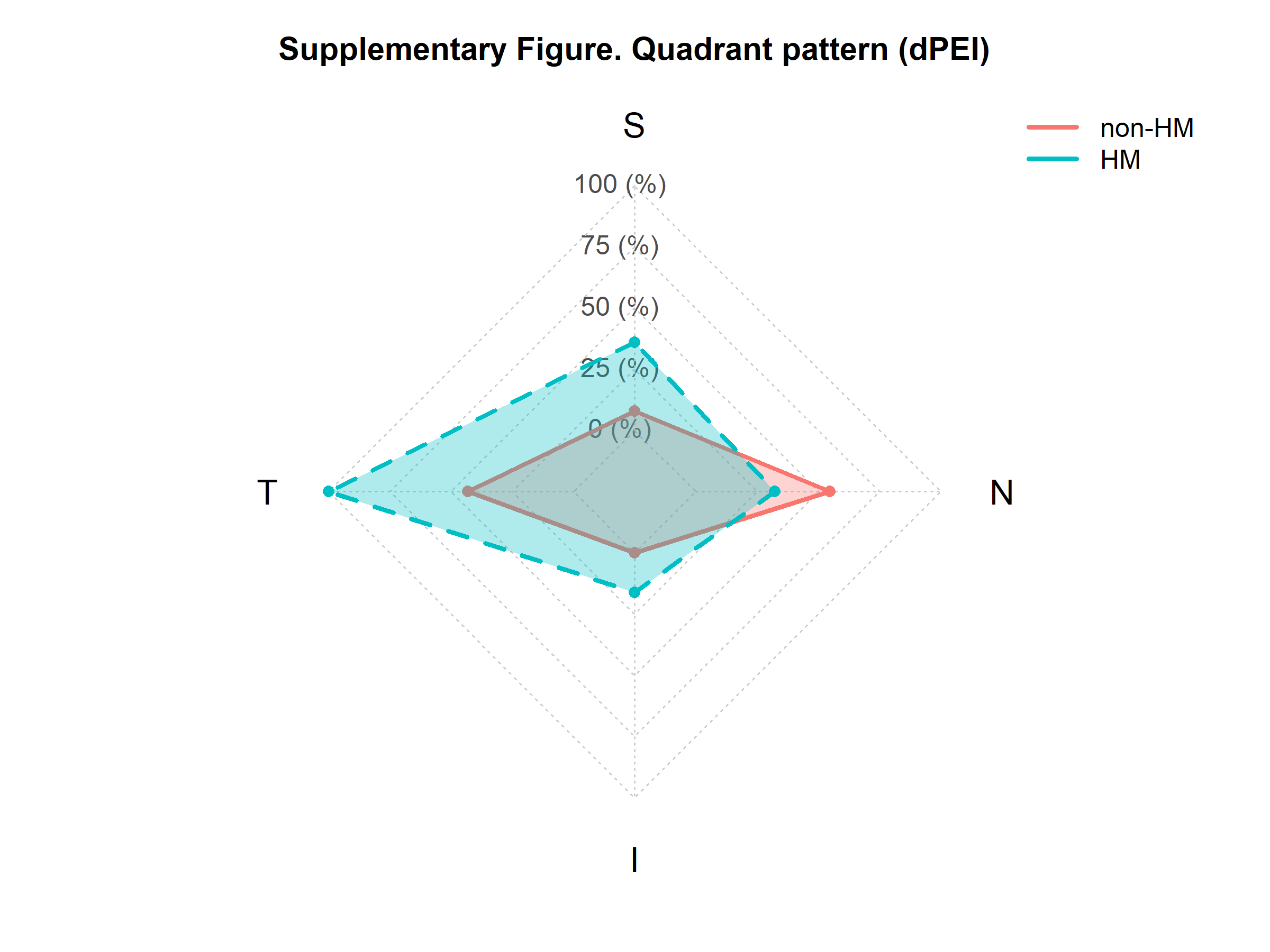


Radar plots display group mean values of quadrant-specific directional posterior expansion indices (dPEI) in the superior (S), inferior (I), nasal (N), and temporal (T) quadrants for high myopia (HM) and non–high myopia (non-HM) eyes. dPEI was calculated using an outer–inner ring difference approach (R6–R2) within each quadrant, such that larger dPEI values indicate greater peripheral expansion relative to the inner macular region.

This figure is intended as a descriptive morphology “pattern” visualization to complement the main inferential analyses, highlighting the directional distribution of posterior pole remodeling rather than emphasizing statistical significance.

Abbreviations: dPEI = directional posterior expansion index; HM = high myopia; N = nasal; non-HM = non–high myopia; S = superior; I = inferior; T = temporal.

**Supplementary Figure S13. Clinically interpretable effect sizes for posterior pole morphology (per +1 mm axial length or per −3 D spherical equivalent)**

**
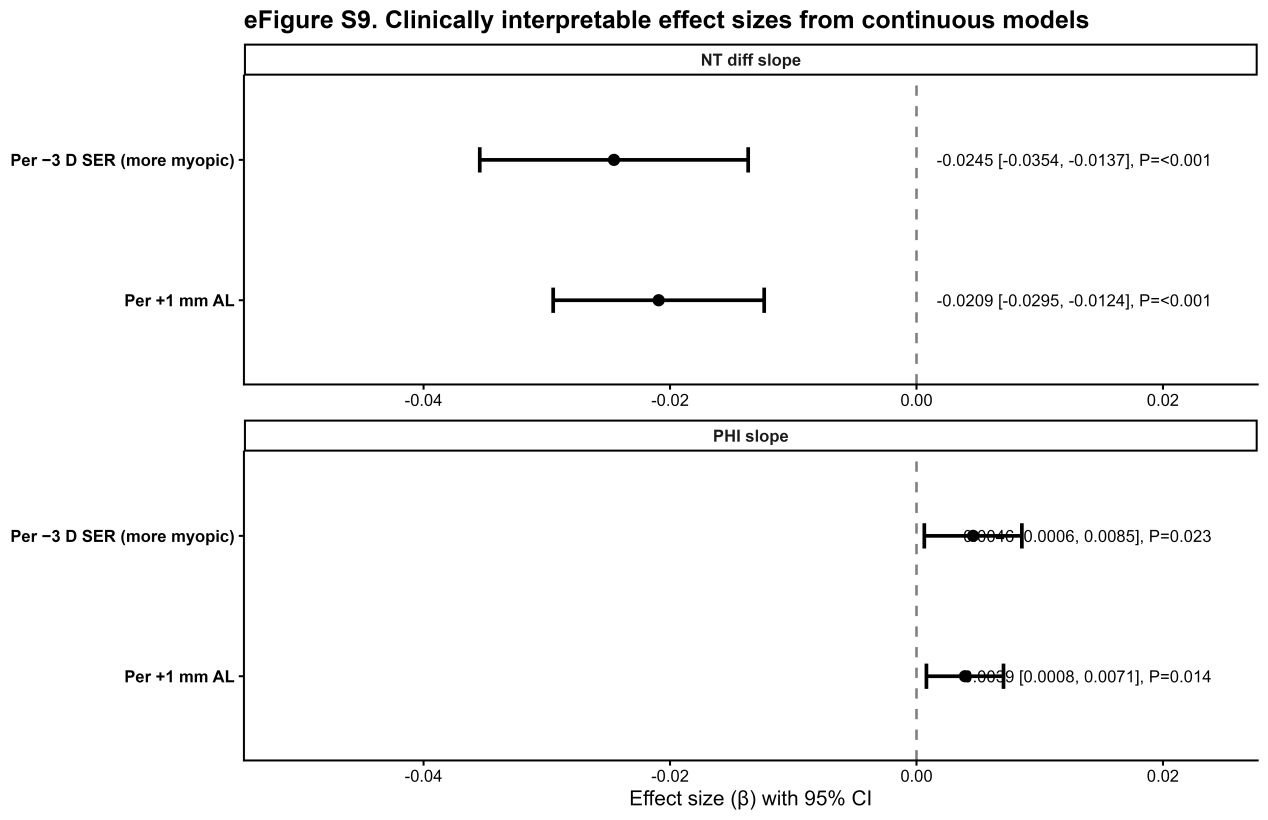
**

Adjusted effect sizes derived from continuous linear mixed-effects models are presented as changes in posterior pole morphology per clinically interpretable units: +1 mm increase in axial length (AL) or −3 diopters (D) decrease in spherical equivalent refraction (SER). Forest plots display β estimates with 95% confidence intervals for PHI slope and NT diff slope. This representation facilitates clinical interpretation by translating statistical associations into commonly encountered refractive and biometric increments.

Abbreviations: AL = axial length; SER = spherical equivalent refraction; PHI = posterior heterogeneity index; NT = nasal–temporal; CI = confidence interval.

**Supplementary Figure S14. Posterior pole morphology across axial length tertiles within high myopia**

Within the high myopia group, eyes were stratified into tertiles based on axial length. Distributions of PHI slope and NT diff slope are shown for each tertile. This descriptive analysis explores whether posterior pole morphology varies with increasing axial elongation among highly myopic eyes, without formal hypothesis testing.


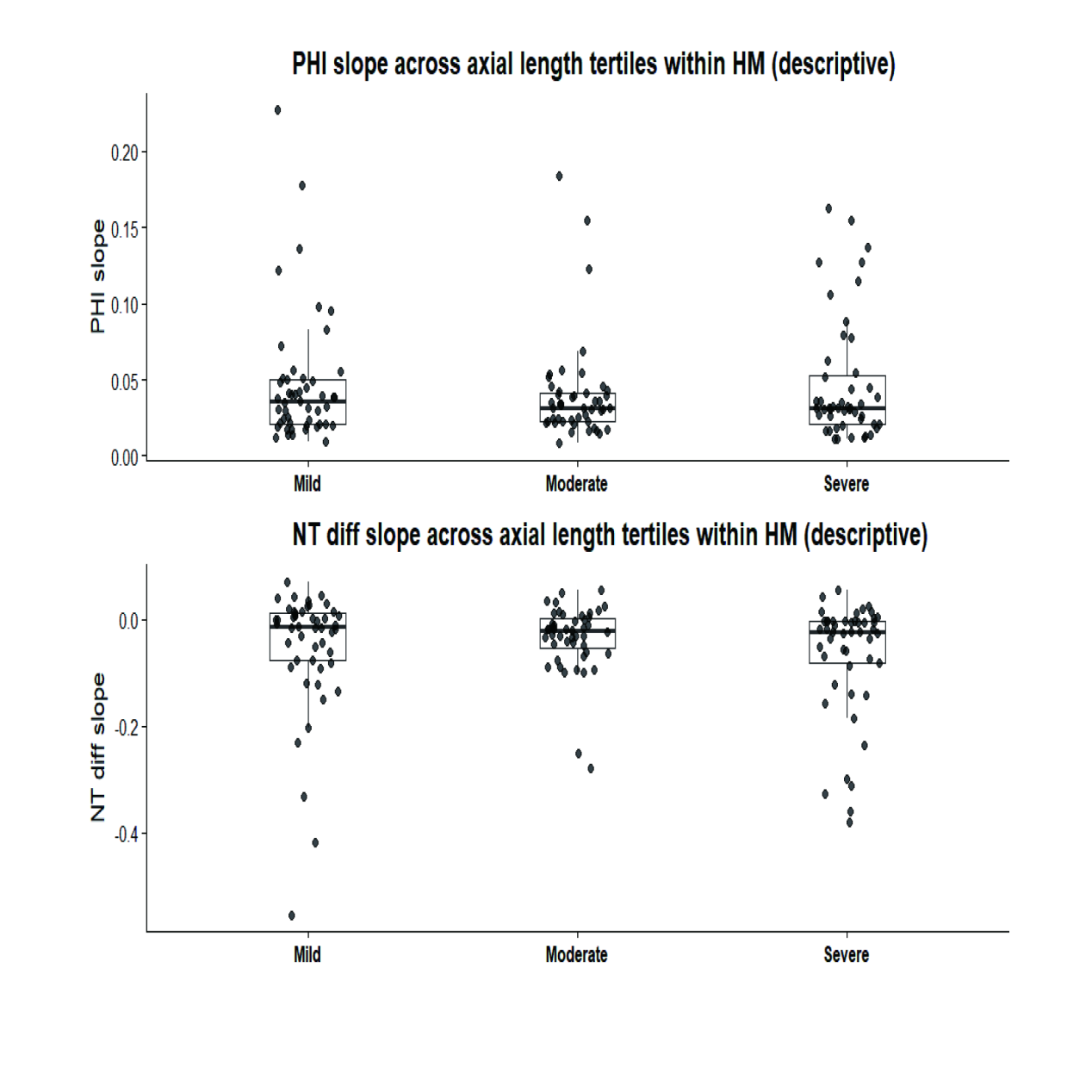


### Abbreviations: AL = axial length; SER = spherical equivalent refraction; HM = high myopia; PHI = posterior heterogeneity index; NT = nasal–temporal.

**Supplementary Figure S15. Posterior pole morphology across spherical equivalent tertiles within high myopia**


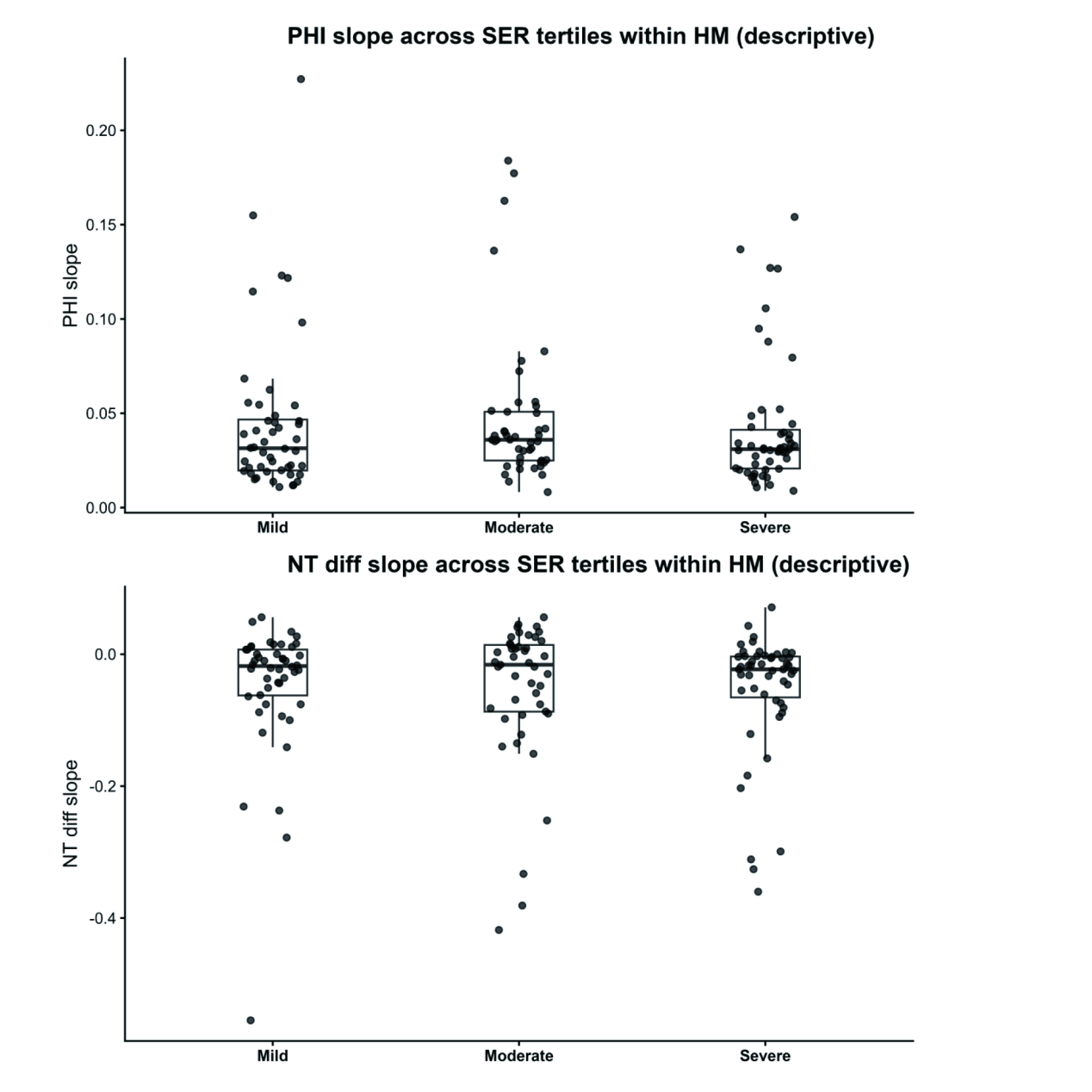

Within the high myopia group, eyes were stratified into tertiles based on spherical equivalent refraction. Distributions of PHI slope and NT diff slope are displayed for each tertile. These plots provide a descriptive view of morphological trends across refractive severity within high myopia.

### Abbreviations: AL = axial length; SER = spherical equivalent refraction; HM = high myopia; PHI = posterior heterogeneity index; NT = nasal–temporal.

**Supplementary Section G — Discordant AL–SER Phenotypes: Structural–Refractive Dissociation as a Key Exploratory Clinical Scenario**

This supplementary section explores a clinically relevant but underrecognized scenario in which axial length (AL) and spherical equivalent refraction (SER) are discordant. By integrating posterior pole morphology into AL–SER phenotyping, we examine whether eyes classified as high myopia by SER but not by AL demonstrate structural features more consistent with high myopia–like remodeling. These analyses are exploratory and aim to illustrate structural–refractive dissociation rather than to establish diagnostic thresholds or predictive models.

**Supplementary Figure S16. Discordant AL–SER phenotype groups: distribution of PHI slope**

**
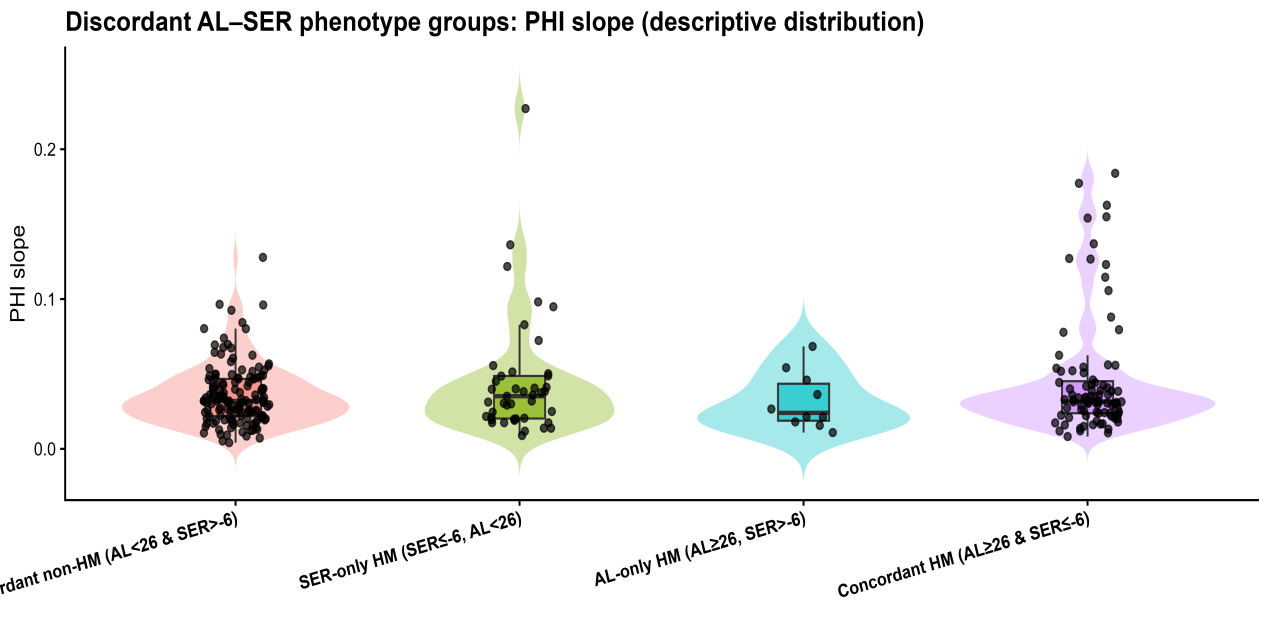
**

Violin and box plots showing the distribution of posterior heterogeneity index (PHI) slope across AL–SER phenotype groups, including concordant non–high myopia, SER-only high myopia, AL-only high myopia, and concordant high myopia. Individual data points represent single eyes. PHI slope reflects posterior pole curvature heterogeneity and is shown for descriptive comparison across phenotype groups.

## **Supplementary Figure S17. Discordant AL–SER phenotype groups: distribution of NT difference slope**


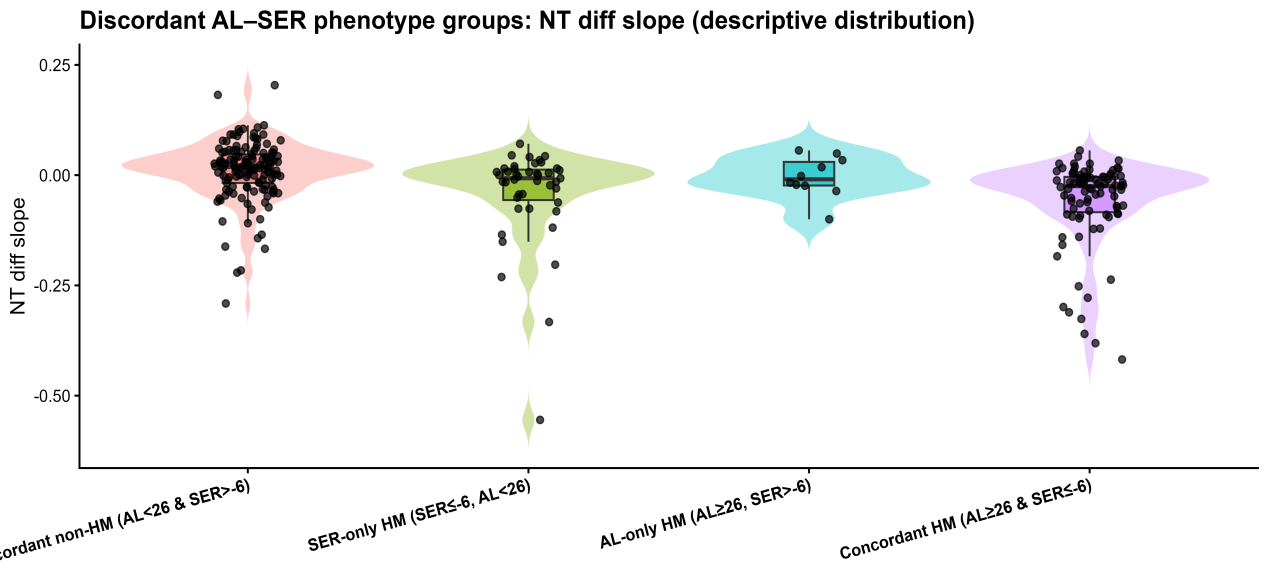
Distribution of nasal–temporal (NT) curvature difference slope across AL–SER phenotype groups. NT difference slope captures directional asymmetry of posterior pole curvature. Violin plots summarize group-level distributions, with overlaid box plots and individual observations.

## **Supplementary Figure S18. Discordant AL–SER phenotype groups: selected supportive posterior pole metrics**


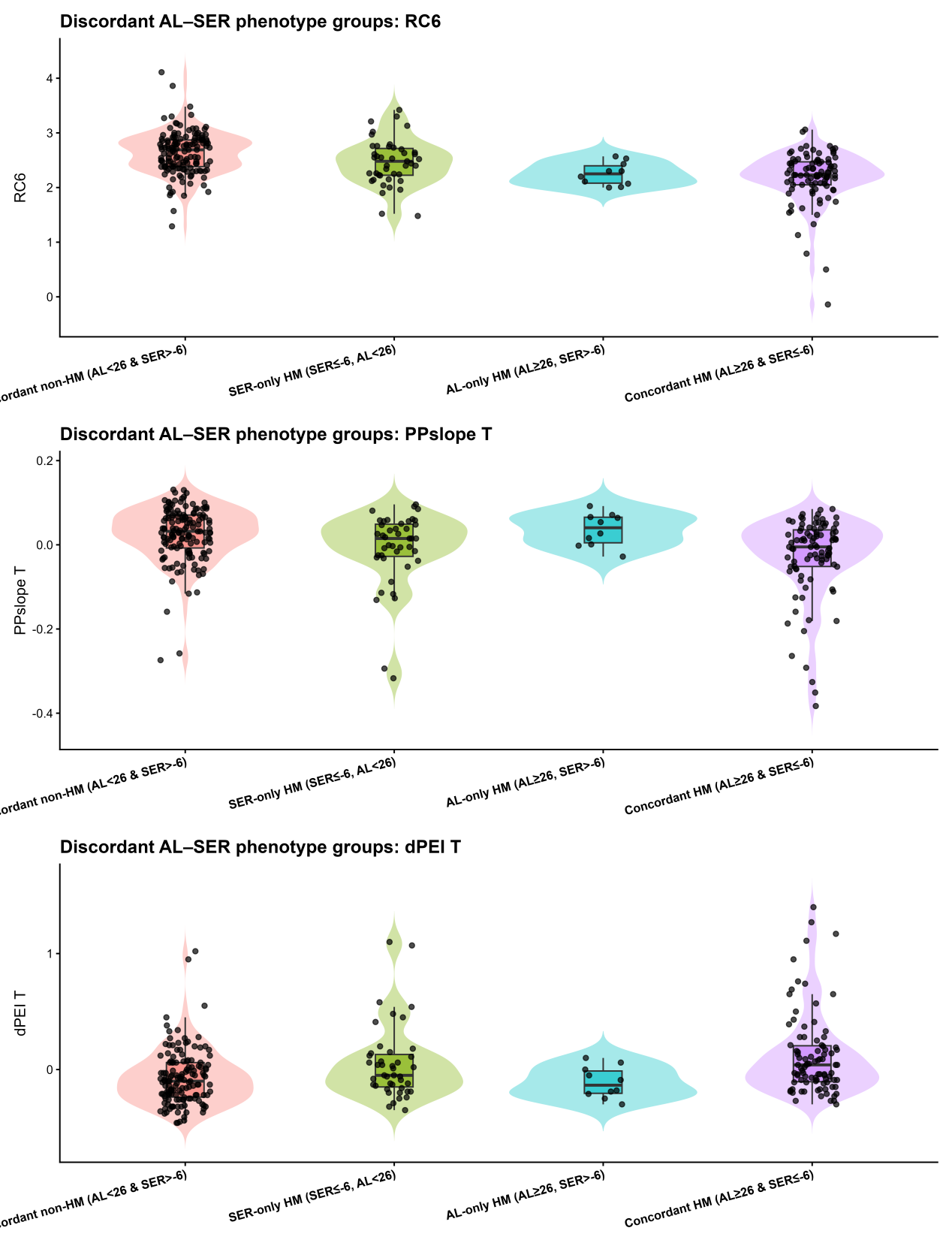


## Distributions of selected secondary posterior pole metrics, including ring-based curvature (RC6), temporal posterior pole slope (PPslope T), and temporal differential posterior ectasia index (dPEI T), across AL–SER phenotype groups. These metrics are presented as supportive descriptors to complement PHI and NT-based analyses.

## **Supplementary Table S4. Linear mixed-effects models comparing posterior pole morphology across discordant AL–SER phenotype groups**

**Panel A. Descriptive summary by phenotype group (eye-level)**

| **Phenotype group** | **eyes** | **subjects** | **AL mean** | **SER mean** | **PHI slope mean** | **NT diff slope mean** |
| --- | --- | --- | --- | --- | --- | --- |
| Concordant non-HM  (AL<26 , SER>-6) | 142 | 77 | 24.458 | -2.798 | 0.036 | 0.010 |
| SER-only HM  (SER≤-6, AL<26) | 43 | 27 | 25.336 | -7.000 | 0.045 | -0.044 |
| AL-only HM (AL≥26, SER>-6) | 10 | 7 | 26.420 | -4.700 | 0.032 | -0.004 |
| Concordant HM (AL≥26 , SER≤-6) | 91 | 52 | 26.991 | -8.692 | 0.046 | -0.060 |

**Panel B. LMM-adjusted group contrasts (reference = Concordant non-HM)**

| **index** | **Contrast (non-HM)** | **β** | **95% CI** | ***P* value** |
| --- | --- | --- | --- | --- |
| PHI slope | SER-only HM | 0.0114 | 0.0003 to 0.0225 | **0.044** |
| PHI slope | AL-only HM | 0.0034 | −0.0167 to 0.0236 | 0.736 |
| PHI slope | Concordant HM | 0.0131 | 0.0037 to 0.0225 | **0.006** |
| NT diff slope | SER-only HM | −0.0504 | −0.0809 to −0.0200 | **0.001** |
| NT diff slope | AL-only HM | −0.0211 | −0.0764 to 0.0343 | 0.454 |
| NT diff slope | Concordant HM | −0.0675 | −0.0933 to −0.0418 | **<0.001** |
| RC6 | SER-only HM | −0.1972 | −0.3533 to −0.0412 | **0.013** |
| RC6 | AL-only HM | −0.3653 | −0.6496 to −0.0810 | **0.012** |
| RC6 | Concordant HM | −0.4561 | −0.5862 to −0.3260 | **<0.001** |
| PPslope T | SER-only HM | −0.0438 | −0.0722 to −0.0153 | **0.003** |
| PPslope T | AL-only HM | −0.0147 | −0.0662 to 0.0368 | 0.575 |
| PPslope T | Concordant HM | −0.0592 | −0.0833 to −0.0350 | **<0.001** |
| dPEI T | SER-only HM | 0.1631 | 0.0588 to 0.2673 | **0.002** |
| dPEI T | AL-only HM | 0.0588 | −0.1303 to 0.2479 | 0.541 |
| dPEI T | Concordant HM | 0.219 | 0.1308 to 0.3072 | **<0.001** |

Results of linear mixed-effects models evaluating differences in posterior pole morphology metrics across AL–SER phenotype groups. Models included group as a fixed effect and were adjusted for age, sex, eye laterality, and study center, with subject-level random intercepts to account for inter-eye dependency. Separate models were fitted for PHI slope, NT difference slope, and selected secondary metrics. Estimates are reported with 95% confidence intervals.

## **Supplementary Figure S19. Axial length–spherical equivalent scatter with continuous PHI slope overlay**


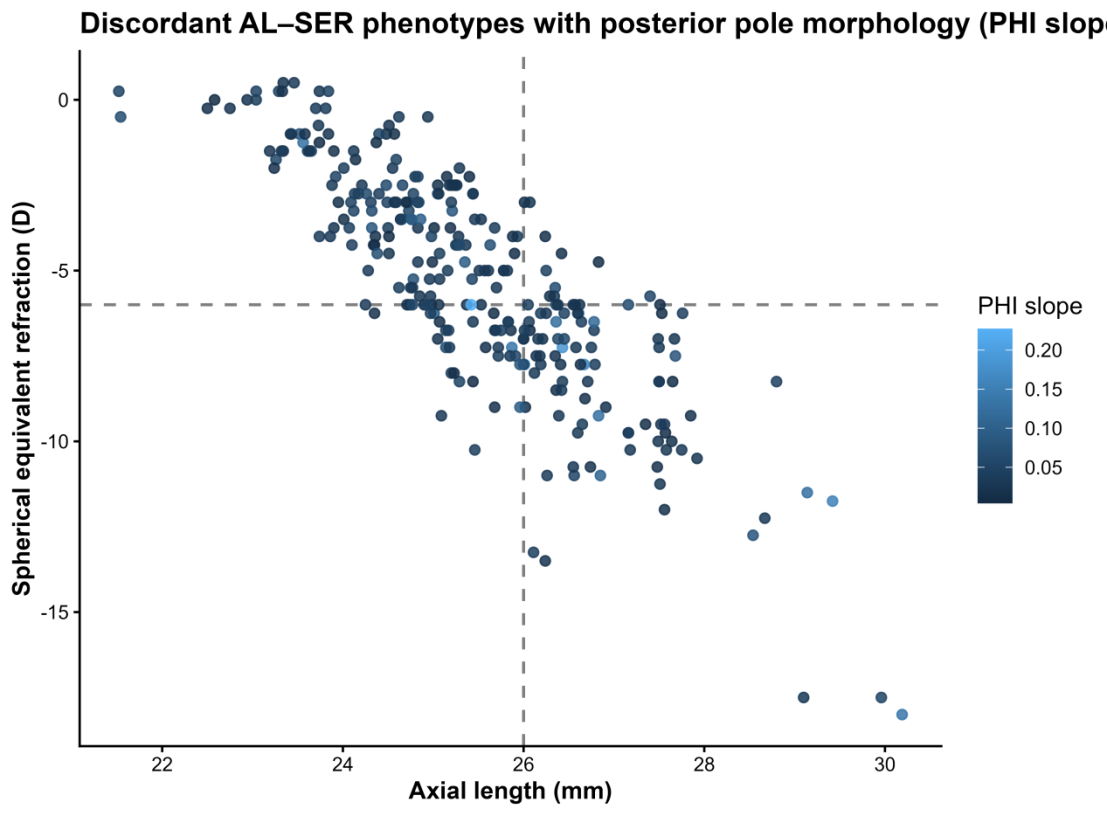


Scatter plot of axial length (AL) versus spherical equivalent refraction (SER), with points colored by continuous PHI slope values. Vertical and horizontal dashed lines indicate conventional thresholds for AL (26 mm) and SER (−6.0 D). This visualization illustrates the distribution of posterior pole heterogeneity across concordant and discordant AL–SER phenotypes.

## **Supplementary Figure S20. Axial length–spherical equivalent scatter with continuous NT difference slope overlay**


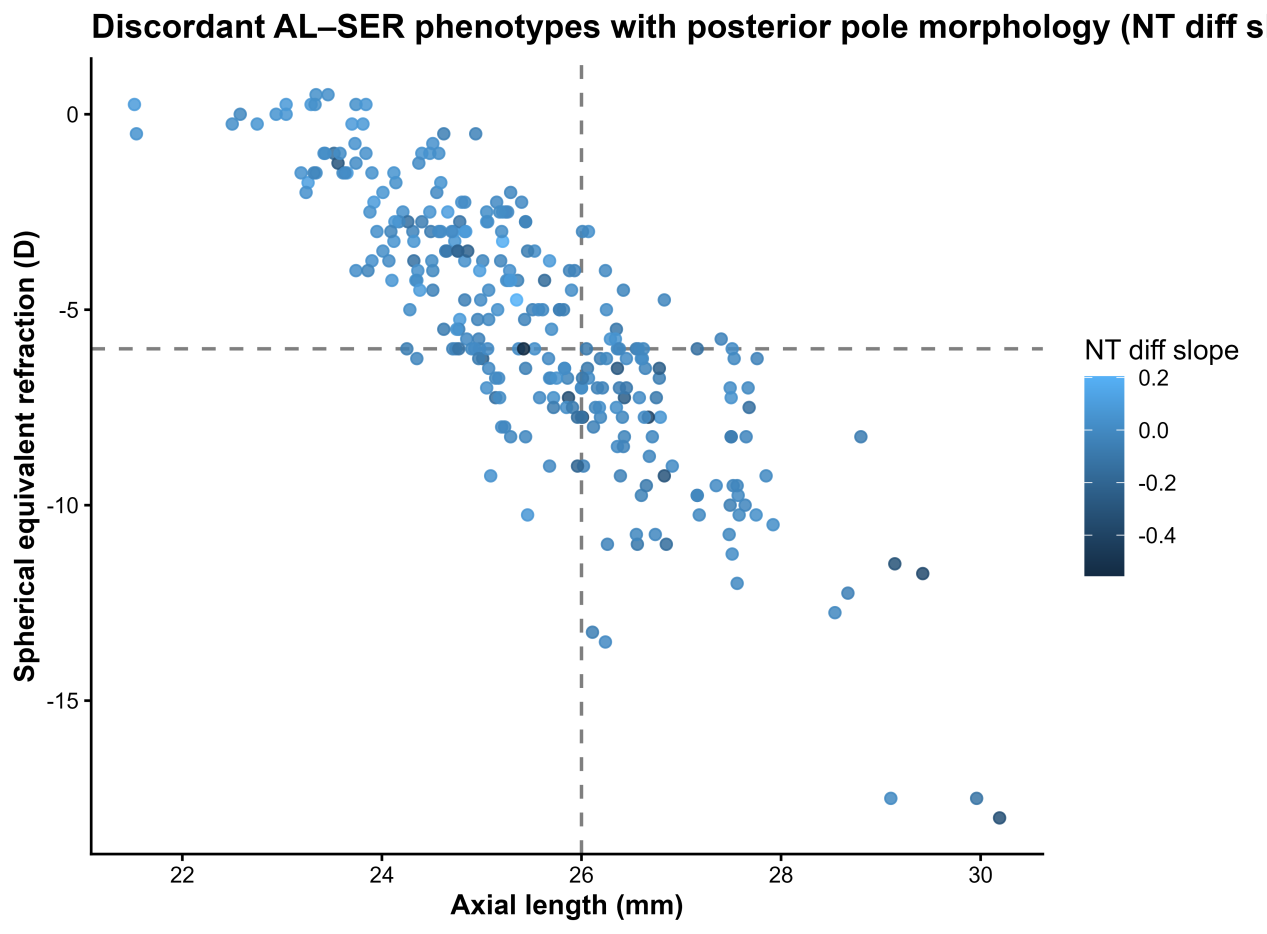


AL–SER scatter plot with points colored according to NT difference slope values. The color gradient reflects the degree of nasal–temporal posterior pole asymmetry. Conventional AL and SER thresholds are shown for reference.

##

## **Supplementary Figure S21. Axial length–spherical equivalent scatter stratified by binary PHI risk (print-friendly)**


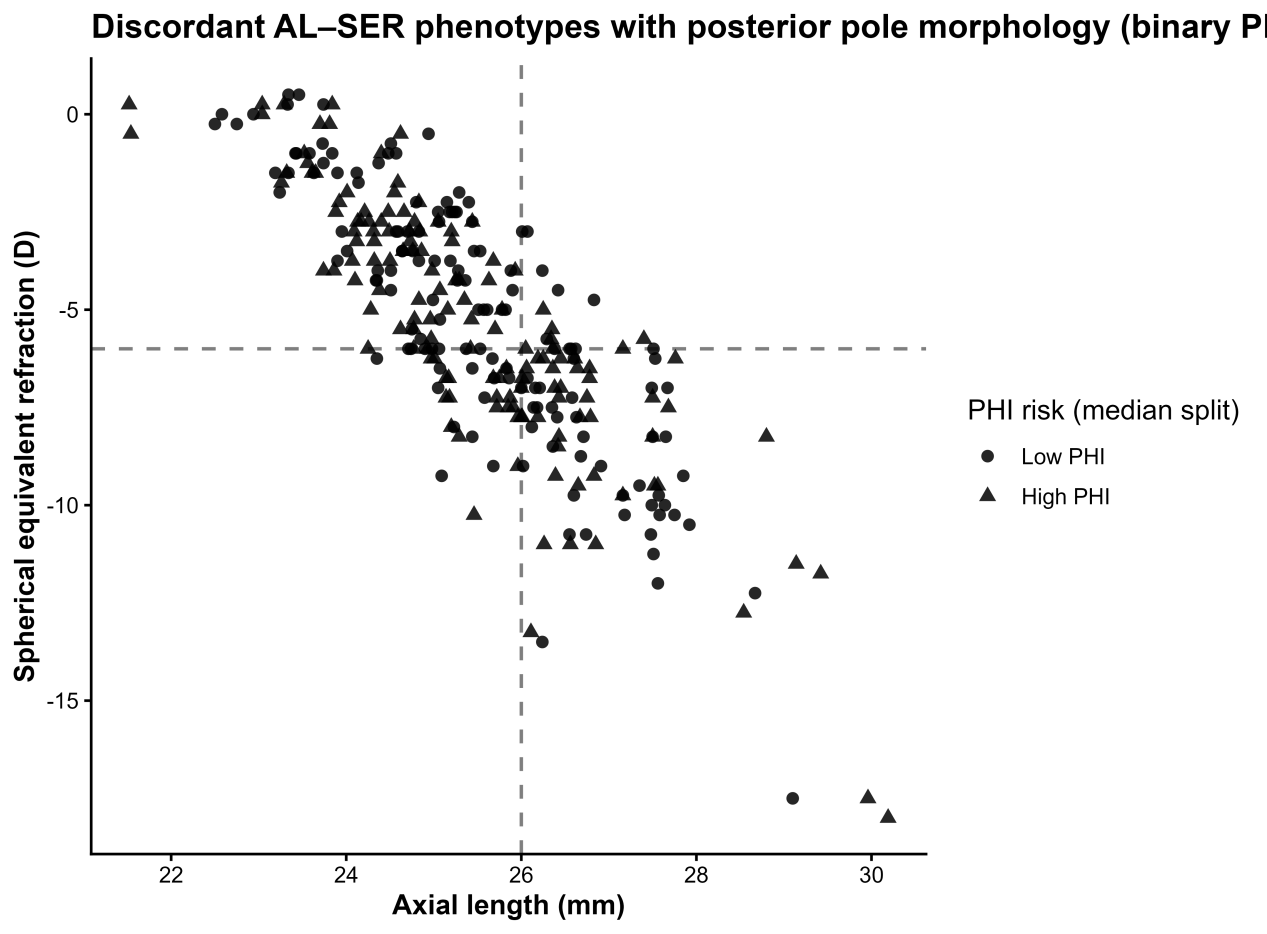
AL–SER scatter plot stratified by binary PHI risk category (high vs low), defined by median split of PHI slope. Point shapes indicate PHI risk group, facilitating print-friendly visualization. This figure is intended for conceptual illustration of structural–refractive mismatch rather than risk stratification.

## **Supplementary Figure S22. Planned contrasts focusing on SER-only high myopia phenotype**


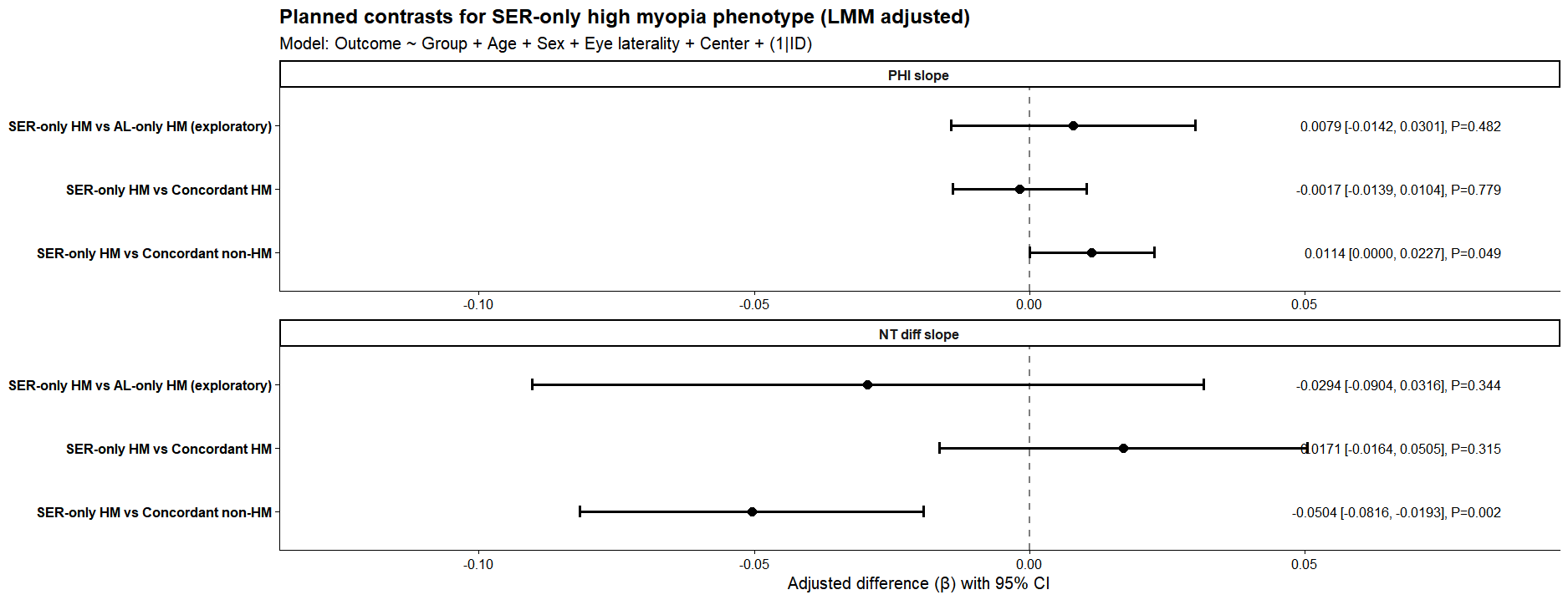
Forest plot of planned contrasts comparing the SER-only high myopia group with concordant non–high myopia, concordant high myopia, and AL-only high myopia groups. Effect estimates are derived from linear mixed-effects models adjusted for age, sex, eye laterality, and study center, with subject-level random intercepts. Results are shown separately for PHI slope and NT difference slope.

Abbreviations: AL = Axial length; SER = Spherical equivalent refraction; HM = High myopia

; PHI = Posterior heterogeneity index; NT = nasal–temporal; RC = Ring curvature; PPslope = Posterior pole slope; dPEI = Differential posterior ectasia index; LMM = Linear mixed-effects model; CI = Confidence interval.

**Supplementary Section H — Exploratory analyses: SER explained by posterior pole morphology beyond axial length**

To explore whether posterior pole morphology provides incremental information beyond axial length (AL) in explaining refractive error, we conducted exploratory analyses modeling spherical equivalent refraction (SER) with and without morphology-related parameters. These analyses were designed to assess explanatory value rather than prediction and should be interpreted as hypothesis-generating. Results from this section do not alter the primary conclusions of the study.

**Supplementary Table S5. Incremental explanatory value of posterior pole morphology for spherical equivalent refraction beyond axial length**

**Panel A. Model fit indices (same sample across models)**

| **Model** | **Formula (core terms)** | **eyes** | **AIC** | **BIC** | **Marginal R²** | **Conditional R²** |
| --- | --- | --- | --- | --- | --- | --- |
| M1 | SER ~ AL + covariates + (1\|ID) | 143 | 286 | 837.04 | 866.28 | 0.7228 |
| M2 | SER ~ AL + PHI slope + NT diff slope + covariates +(1\|ID) | 143 | 286 | 840.27 | 876.83 | 0.7219 |
| M3 | SER ~ AL + PHI slope + NT diff slope + (1–3 extra morphology) + covariates + (1\|ID) | 143 | 286 | 839.68 | 887.21 | 0.7214 |

**Panel B. Key fixed-effect coefficients**

| **Model** | **Term** | **β** | **95% CI** | ***P* value** |
| --- | --- | --- | --- | --- |
| M1 | AL | −2.0237 | −2.1654 to −1.8820 | **<0.001** |
| M2 | AL | −2.0342 | −2.1775 to −1.8910 | **<0.001** |
| M2 | PHI slope | 0.6843 | −3.1505 to 4.5190 | 0.725 |
| M2 | NT diff slope | −0.3729 | −1.7377 to 0.9920 | 0.59 |
| M3 | AL | −2.0388 | −2.1926 to −1.8851 | **<0.001** |
| M3 | PHI slope | 1.014 | −2.9986 to 5.0266 | 0.618 |
| M3 | NT diff slope | −1.9016 | −3.8974 to 0.0942 | 0.062 |

**Panel C. Grouped 5-fold cross-validation (subject-level grouped CV)**

| **Model** | **k-fold** | **eyes** | **RMSE mean** | **RMSE SD** | **MAE mean** | **MAE SD** |
| --- | --- | --- | --- | --- | --- | --- |
| M1 | 5 | 286 | 1.7751 | 0.2527 | 1.3961 | 0.1584 |
| M2 | 5 | 286 | 1.7834 | 0.2475 | 1.4035 | 0.1548 |
| M3 | 5 | 286 | 1.774 | 0.2506 | 1.3995 | 0.1517 |

Linear mixed-effects models were fitted with SER as the dependent variable. Model 1 included axial length and clinical covariates only, while Model 2 additionally incorporated posterior pole morphology parameters (PHI slope, NT diff slope, and selected secondary metrics). Model performance was compared using marginal R², information criteria, and cross-validated prediction error. These analyses were exploratory and intended to assess incremental explanatory value rather than clinical prediction. Overall, adding posterior pole morphology metrics did not meaningfully improve model fit or cross-validated error beyond AL, suggesting limited incremental explanatory value for SER in this cohort.

**Supplementary Table S6. Continuous linear mixed-effects models using spherical equivalent refraction as the exposure variable**

| **index** | **eyes** | **β (95% CI)** | ***P* value** |
| --- | --- | --- | --- |
| PHI slope | 286 | -0.0015 [-0.0028, -0.0002] | **0.023** |
| NT diff slope | 286 | 0.0082 [0.0046, 0.0118] | **<0.001** |

To evaluate whether associations observed using axial length were robust to alternative exposure specification, linear mixed-effects models were refitted with SER as the primary exposure variable. Outcomes included posterior pole morphology parameters. These models serve as sensitivity analyses and are not intended for causal inference.

Abbreviations: AL = axial length; SER = spherical equivalent refraction; PHI = posterior heterogeneity index; NT diff = nasal–temporal curvature difference; LMM = linear mixed-effects model; RMSE = root mean squared error; MAE = mean absolute error.

# ****Supplementary Section I — Conceptual schematic**** This section presents a conceptual, non-predictive schematic illustrating how posterior pole morphology may complement conventional axial length (AL) and spherical equivalent refraction (SER)–based assessment. The schematic is intended to enhance clinical interpretability, particularly in eyes with discordant AL–SER phenotypes, by providing an additional structural context rather than redefining high myopia or offering risk thresholds. This framework is hypothesis-generating and designed for research interpretation only.

**Supplementary Figure S23. Conceptual decision-support schematic illustrating how posterior pole morphology complements conventional axial length (AL) and spherical equivalent refraction (SER) assessment.**


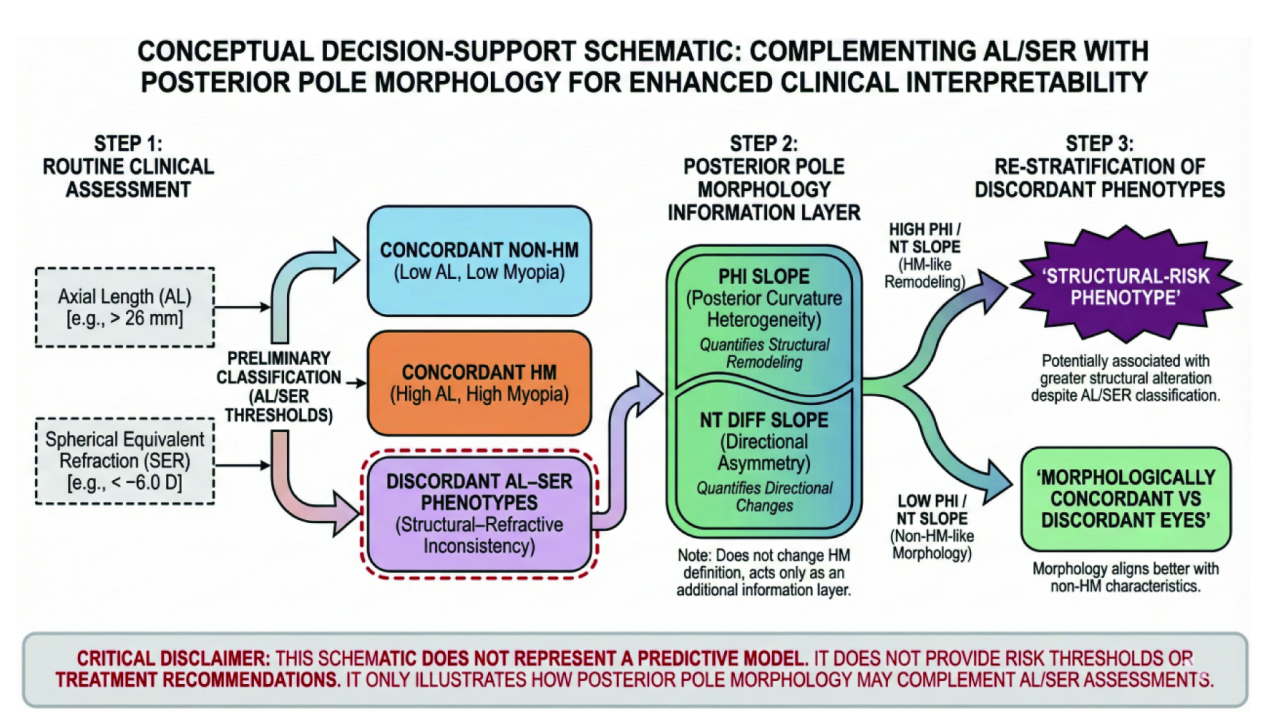

The schematic outlines a three-step interpretative framework. Step 1 shows routine clinical classification based on established AL and SER thresholds, categorizing eyes into concordant non–high myopia (non-HM), concordant high myopia (HM), or discordant AL–SER phenotypes. Step 2 introduces posterior pole morphology metrics—PHI slope (posterior curvature heterogeneity) and NT diff slope (directional asymmetry)—as an additional information layer that quantifies structural remodeling without altering HM definitions. Step 3 illustrates the re-stratification of discordant phenotypes into morphology-informed subgroups, including an HM-like “structural-risk phenotype” and a non-HM–like morphological pattern. This schematic does not represent a predictive model, does not define risk cutoffs, and does not provide treatment recommendations. It is intended solely to illustrate how posterior pole morphology may enhance the interpretability of AL/SER-based classifications in a research context.

Abbreviations: AL = axial length; SER = spherical equivalent refraction; HM = high myopia; PHI = posterior heterogeneity index; NT diff = nasal–temporal difference; LMM = linear mixed-effects model.
