# Supplementary material for: Asymmetry of posterior pole remodeling in high myopia
Source: Sci Rep. 2026 Apr 21;16:18586. doi: 10.1038/s41598-026-49683-w (PMC13269795; doi:10.1038/s41598-026-49683-w)
Supplement: Supplementary file 2 — Supplementary Material 2 [file 41598_2026_49683_MOESM2_ESM.docx]

**Graphical Abstract. Asymmetry of Posterior Pole Remodeling in High Myopia**


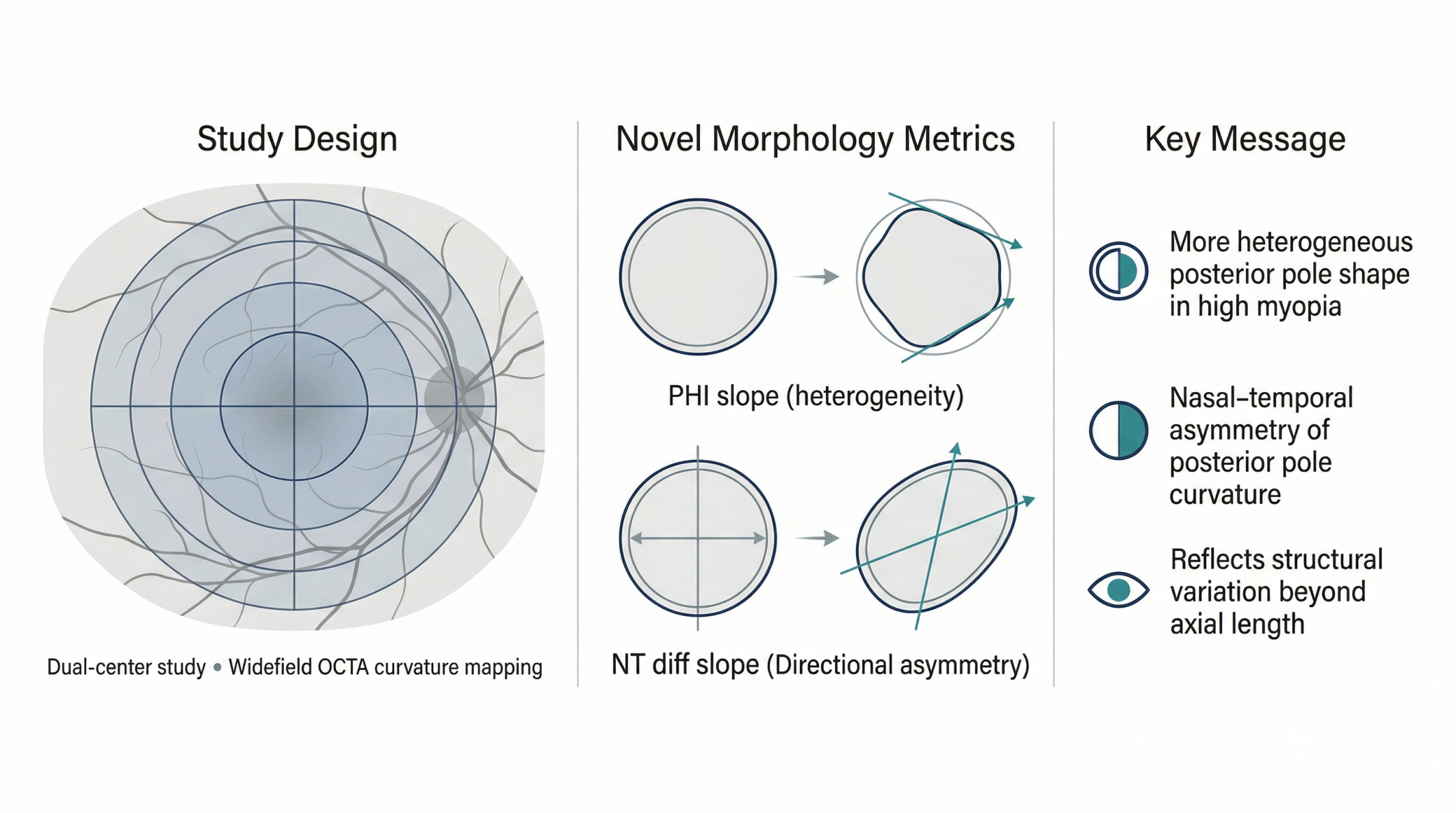


Graphical abstract summarizing the study design and main findings: widefield OCTA–derived curvature mapping quantifies posterior curvature heterogeneity (PHI slope) and nasal–temporal asymmetry (NT diff slope), showing asymmetric posterior pole remodeling in high myopia beyond axial length alone.

Abbreviations: NT = nasal–temporal; OCTA = optical coherence tomography angiography; PHI = posterior heterogeneity index.

Note: This graphical abstract is conceptual and does not represent a predictive model or provide clinical risk thresholds.
